# Microstructure-informed deep learning improves thalamic atrophy segmentation and clinical associations in multiple sclerosis and related neuroimmunological diseases

**DOI:** 10.1016/j.nicl.2026.103982

**Published:** 2026-03-02

**Authors:** Omar Angelo Ibrahim, Henri Trang, Qianlan Chen, Lara Zimmermann, Alexander U. Brandt, Tatiana Usnich, Stefano Magon, Muhamed Barakovic, Jens Wuerfel, Friedemann Paul, Martin Bauer, Lina Anderhalten

**Affiliations:** a Experimental and Clinical Research Center (ECRC), A Cooperation Between Max Delbrück Center for Molecular Medicine in the Helmholtz Association and Charité – Universitätsmedizin Berlin, Lindenberger Weg 80, 13125 Berlin, Germany; b Max Delbrück Center for Molecular Medicine in the Helmholtz Association (MDC), Robert-Rössle-Straße 10, 13125 Berlin, Germany; c F. Hoffmann-La Roche Ltd, Grenzacherstrasse 124, CH-4070 Basel, Switzerland; d Neuroscience Clinical Research Center (NCRC), Charité - Universitätsmedizin Berlin, Corporate member of Freie Universität Berlin, Humboldt-Universität zu Berlin, Charitéplatz 1, 10117 Berlin, Germany; e Department of Neurology with Experimental Neurology, Charité - Universitätsmedizin Berlin, Charitéplatz 1, 10117 Berlin, Germany; f Charité - Universitätsmedizin Berlin, Corporate Member of Freie Universität Berlin and Humboldt-Universität zu Berlin, Department of Medical Psychology, Charitéplatz 1, 10117 Berlin, Germany; g Department of Neurology, University of California, Irvine School of Medicine, Orange, CA, United States

**Keywords:** Multiple sclerosis, Neuromyelitis-optica spectrum disorder, Myelin oligodendrocyte glycoprotein antibody-associated disease, Quantitative magnetic resonance imaging, Multi-parameter mapping, 3D convolutional neural network, Thalamus

## Abstract

Thalamic atrophy is a sensitive imaging marker of neurodegeneration in multiple sclerosis (MS) and related disorders, though thalamus segmentation remains method-dependent. Quantitative magnetic resonance imaging (qMRI) may enhance thalamic boundary contrast, particularly in the context of deep learning. We benchmarked thalamic segmentations from two atlas-constrained algorithms, FreeSurfer and FIRST, and two deep learning algorithms, DBSegment and MindGlide (an MS-trained model), against ground truth (GT) labels, tested whether quantitative R1 maps improve performance, and evaluated clinical validity cross-sectionally and longitudinally. We generated thalamus masks using each algorithm from T1-weighted data in a single-scanner cohort (baseline n = 321; 1-year follow-up n = 234) including patients with MS/related disorders and healthy controls. Using MindGlide, we also produced FLAIR- and R1-based masks and ensembles. Manual GT labels were obtained for 50 MS patients using T1w and FLAIR scans. For voxel-wise GT agreement, DBSegment yielded the highest Dice-similarity coefficient; atlas-constrained methods showed the highest sensitivity but lowest precision, while MindGlide balanced both. Volumetrically, MindGlide showed the most accurate estimates; DBSegment and FreeSurfer showed proportional bias, and both atlas-constrained methods overestimated thalamic volumes. Adding R1 input to MindGlide produced modest or no gains in GT agreement. Additionally, MindGlide volumes were most consistently associated with disability and cognitive scores cross-sectionally, and longitudinally showed the largest effects between thalamic volume change and EDSS worsening. Incorporating R1 maps offered no cross-sectional benefit but strengthened longitudinal associations. Higher-resolution qMRI and multi-contrast deep learning architectures may further enhance thalamic segmentation and monitoring in neuroinflammatory diseases.

## Highlights

• Deep learning improved thalamus segmentation in multiple sclerosis brain scans.
• Atlas based methods overestimated thalamus volume despite spatial overlap.
• Voxel overlap and volume accuracy diverged across segmentation tools.
• Quantitative magnetic resonance maps modestly improved disability associations.

## 1 Introduction

Multiple sclerosis (MS) is a chronic autoimmune disease of the central nervous system (CNS), that affects about 2.8 million people worldwide (Portaccio et al., 2024). Clinically, MS is categorized into different subtypes: Relapsing-remitting MS (RRMS), which accounts for about 85% of diagnoses and is defined by acute relapses with partial or full recovery; and progressive MS (PMS), including primary and secondary PMS, which are marked by gradual, relapse-independent neurological decline (McGinley et al., 2021, Thompson et al., 2018). Nevertheless, recent evidence increasingly supports a view of MS as a pathological continuum, where neurodegenerative processes occur across all disease stages and phenotypes (Kuhlmann et al., 2023), with disability progression often occurring independent of relapse activity (PIRA) (Tur et al., 2023). Thalamic atrophy, an emerging MRI-derived biomarker for neurodegeneration in MS (Azevedo et al., 2018, Stankoff and Louapre, 2018, Krijnen et al., 2023), can be detected already in early stages, including the Radiologically Isolated Syndrome (RIS) (Azevedo et al., 2015) and the Clinically Isolated Syndrome (CIS) (Kipp et al., 2015, Tommasin et al., 2021). Additionally, thalamic atrophy has been observed in rarer neuro-immunological diseases such as Neuromyelitis-Optica Spectrum Disorder (NMOSD) (Hyun et al., 2017, Seok et al., 2022, Alosaimi et al., 2024) and Myelin Oligodendrocyte Glycoprotein Antibody-associated Disease (MOGAD) (Cacciaguerra et al., 2025, Rechtman et al., 2022, Zhuo et al., 2021). The clinical relevance of thalamic atrophy in MS patients is well established (Amin and Ontaneda, 2020). It has been identified as a major driver of disability progression (Eshaghi et al., 2018, Hänninen et al., 2020); across cohorts, it correlated strongly with the Expanded Disability Status Scale (EDSS) score (Hänninen et al., 2020, Minagar et al., 2013, Eshaghi et al., 2018), and has been reported to outperform whole-brain atrophy for predicting long-term disability (Hänninen et al., 2020). In addition, it has been consistently associated with cognitive impairment in CIS (Amin and Ontaneda, 2020, Štecková et al., 2014, Paul, 2016) and in RRMS, independent of other clinical features (Lorefice et al., 2020). Disease-modifying therapies attenuate thalamic volume loss (Azevedo et al., 2018, Raji et al., 2018, Gaetano et al., 2018, Arnold et al., 2022, Marastoni et al., 2022), supporting its use as an endpoint in clinical trials (Schoonheim and Ciccarelli, 2018). In this context, treatment with ocrelizumab, compared to IFNβ1a/placebo, has been observed to be associated with lower thalamic atrophy, which was in turn linked to better clinical outcomes of disability in MS (Arnold et al., 2022).

Despite its strong potential as an imaging biomarker of neurodegeneration in MS and other related disorders, thalamic volumetry faces technical challenges. The thalamus is a relatively small and internally heterogeneous structure, and its segmentation has been reported to be sensitive to image resolution, scan quality, and protocol/scanner differences, limiting cross-site comparability (Schwartz et al., 2019). Partial volume effects at the cerebrospinal fluid (CSF)-gray matter (GM) interface and along intra-thalamic boundaries can further complicate accurate thalamic delineation (Tohka, 2014, Bonnier et al., 2016). At the pathophysiological level, degeneration of thalamic projections from widespread white-matter (WM) damage, microglial alterations (Rodriguez-Mogeda et al., 2025), iron-mediated oxidative injury (Pontillo et al., 2021), and deep gray matter (DGM) lesions (Carolus et al., 2022) have been proposed as contributors to thalamic atrophy in MS (Mahajan et al., 2020). Commonly used segmentation automated approaches, such as FreeSurfer (Fischl et al., 2002), which rely on atlas-based tissue priors derived from healthy controls (HC), may therefore introduce bias when applied to thalami with altered contrast from inflammation, demyelination, iron deposition, and atrophy. Apparent volume change may also be confounded by inflammatory swelling (Zivadinov et al., 2008) or by ventricular enlargement (Millward et al., 2020, Sinnecker et al., 2020), particularly in patients with advanced tissue loss. Consistent with these issues, a recent study comparing several segmentation approaches reported systematic overestimation of thalamic volume in MS relative to HC, plausibly due to mismatched tissue priors (Burggraaff et al., 2021).

Taken together, the interpretability of standard volumetry and atrophy measurements may be constrained by disease-impacted thalamic microstructural integrity; microstructure-informed measurement tools are therefore needed. Quantitative MRI (qMRI) offers biophysically informative contrasts and improved reproducibility across time/sites (Weiskopf et al., 2013, Tabelow et al., 2019, Lorio et al., 2016, Lommers et al., 2019, Lommers et al., 2021). For instance, longitudinal relaxation rate (R1) mapping within the multi-parameter mapping (MPM) framework can index myelin-related macromolecular content and iron-sensitive tissue properties. We previously established a 7-minute 3D multi-echo FLASH MPM protocol for whole-brain imaging at 1.6 mm isotropic resolution, well suited for longitudinal clinical studies (Cooper et al., 2020, Trang et al., 2024). In this context, qMRI may help improve thalamic segmentation when used alongside conventional T1-weighted (T1w) MRI, particularly when combined with algorithms trained on MS rather than HC brains. Relatedly, deep learning approaches such as 3D convolutional neural networks (3D-CNN) are increasingly applied to medical image segmentation (Tiwari et al., 2023) due to reduced pre-processing, faster runtimes, and improved performance compared to traditional atlas-based pipelines (Donnay et al., 2023, Goebl et al., 2025, Baniasadi et al., 2023). Combining biologically meaningful qMRI inputs with disease-specific deep learning models may therefore provide the most robust framework for thalamic segmentation in neuroinflammatory diseases. Here, we aimed to (i) benchmark thalamus segmentation performance of two standard atlas-based tools, FreeSurfer (Fischl et al., 2002) and FIRST (Patenaude et al., 2011), and two 3D CNNs, DBSegment (Baniasadi et al., 2023) and MindGlide (Goebl et al., 2025), the latter trained on MS brains (Goebl et al., 2025), against a manual ground truth (GT), and (ii) investigate whether incorporating MPM-derived R1 maps improves segmentation performance and strengthens cross-sectional and longitudinal associations with clinical outcomes.

## 2 Materials and methods

### 2.1 Study design

This retrospective data analysis builds on the BERLimmun (Berlin Registry of Neuroimmunological entities, DRKS00026761) study (Sperber et al., 2022), a single-center prospective observational study approved by the institutional ethics committee under IRB ID EA1/362/20 and conducted in accordance with the 1944 Declaration of Helsinki in its current version for the conduction of the study. Prior to study participation, all participants gave written informed consent. Patient recruitment started at the end of 2021 at the outpatient clinic of the Neuroscience Clinical Research Center at Charité – Universitätsmedizin Berlin and is still ongoing. Inclusion criteria were the following: older than 18 years of age, with active health insurance, and competent to give written informed consent. Exclusion criteria consisted of contraindication to MRI investigation, pregnancy, or diseases hindering the conduct of the study. After the baseline visit, participants were followed up annually. In this retrospective analysis, we included patients with confirmed diagnoses of MS (RRMS and PMS) (Thompson et al., 2018), isolated syndromes (RIS, CIS and isolated Optic Neuritis; iON), aquaporin-4 immunoglobulin G (AQP4-IgG)-seropositive and −negative NMOSD (Wingerchuk et al., 2015), and MOGAD (Banwell et al., 2023), as well as HC, who were enrolled between study initiation and the end of 2024, with at least one available MRI scan at baseline.

### 2.2 Study populations

#### 2.2.1 Full analysis set (FAS)

For cross-sectional analysis of thalamus volume in relation to clinical outcomes (see section 2.3), all participants from BERLimmun with T1w, FLAIR, and R1 data available at study baseline were included in the cross-sectional FAS. This resulted in a full baseline analysis set of n = 321 participants, after the exclusion of 4 participants due to failed R1 map co-registration (see 2.5.2). For longitudinal analysis of thalamic volume loss in relation to changes in clinical outcomes over time, only participants with baseline and 1-year follow-up scans available were included, resulting in a total of n = 234 participants.

#### 2.2.2 Ground truth (GT) analysis set

As manual segmentation is a time-consuming task, baseline scans of n = 50 MS patients were selected for GT analysis. As RRMS was the predominant diagnosis in the cross-sectional FAS, we assembled the GT subset via stratified random sampling of RRMS participants. To generate a representative patient selection, three sampling criteria based on distribution of min, max and mean values in the FAS population were applied: 1) Age group (18–30, 30–50, and 50–70 years old), 2) disease duration, defined as time in years from first symptom onset to the visit date (0–5, 5–20 and 20–40 years) and 3) EDSS score (0–1, 1–3, 3–7). Sampling using R software yielded 34 RRMS participants. Thereafter, distributions across categories were visually assessed using histograms, which closely matched those of the RRMS patients in the cross-sectional FAS (see supplementary Fig. S1, SM.A). Furthermore, all available PMS participants (8 males, 8 females) were added to diversify the dataset, resulting in a total of 25 female and 25 male participants in the GT population.

### 2.3 Clinical assessment of participants

Demographic and clinical parameters collected for study participants included age, sex, disease duration, number of attacks, EDSS score, Timed 25 Foot Walk test (T25FW, in seconds), Nine-Hole Peg Test (9-HPT, in seconds), and Symbol Digit Modalities Test (SDMT, sum of correct answers in 90 s). These clinical outcome measures were selected based on their evidence in the context of thalamic integrity alterations (18–22, 25). T25FW and 9HPT tests as part of the Multiple Sclerosis Functional Composite (MSFC) were performed twice, and results were averaged. All clinical examinations were performed by trained study personnel under the supervision of board-certified neurologists. Longitudinal changes (Δ) in clinical outcome variables (e.g., SDMT and EDSS) were calculated for each participant as the score at the 1-year follow-up minus the baseline score.

### 2.4 MRI acquisition

Sagittal 3D MRI scans were acquired on a 3 T MR scanner (Magnetom Prisma, Siemens Healthineers, Erlangen, Germany) using a 64-channel receive radiofrequency (RF) head-neck coil covering the brain and cervical spinal cord. The acquisition protocol and participant positioning were identical to those detailed in our previous studies (Cooper et al., 2020, Trang et al., 2024). In brief, MPM data was acquired at 1.6 mm isotropic resolution with a field-of-view (FOV) of 224 × 256 mm2 (matrix-size 140 × 160) and involved three distinct 3D multi-echo fast low-angle shot (FLASH) gradient-echo acquisitions, including T1w (TR = 18 ms, 1 min 44 sec), proton density (PD)-weighted (PDw; TR = 18 ms, 1 min 44 sec), as well as magnetization transfer (MT)-weighted (MTw; TR = 37 ms, 3 min 34 sec), with six echos between 2.46 and 14.78 ms, respectively. To enable bias field correction, an RF transmit (B1 + ) map was acquired (2 min 14 sec) for all runs with an isotropic resolution of 4 mm (Cooper et al., 2020, Trang et al., 2024). In addition, the MRI protocol included a high-resolution structural T1w scan (3D MPRAGE, TR = 2,500 ms, TE = 2.22 ms, TI = 1,000 ms, 0.8 mm isotropic resolution), a T2w scan (3D T2 SPACE, TR = 3,200 ms, TE = 563 ms, 0.8 mm isotropic resolution), and T2-weighted fluid-attenuated inversion recovery (3D FLAIR, TR = 6,000 ms, TE = 387 ms, TI = 2,100 ms, 0.8 mm isotropic resolution).

### 2.5 MRI pre-processing

#### 2.5.1 Quantitative map reconstruction

Quantitative map reconstruction followed our previous work (Cooper et al., 2020, Trang et al., 2024). Maps of PD, MTsat, R1, and the effective transverse relaxation rate R2* were generated with the hMRI toolbox (*41*) implemented within SPM12 (http://www.fil.ion.ucl.ac.uk/spm/software/spm12/) using MATLAB (MathWorks, version number 2022b). In brief, quantitative parameters were derived using the ESTATICS model (Weiskopf et al., 2013) from PDw, MTsat, and T1w echoes acquired through FLASH acquisitions (Helms et al., 2008). Prior to parameter quantification, Gibbs-ringing artifacts were removed from all six echoes of the raw images (PDw, MTsat, T1w) (Kellner et al., 2016). Transmit RF field (B1+) imperfections were corrected using the acquired B1 + map within the hMRI toolbox, while receive-field inhomogeneities were corrected using Unified Segmentation (Tabelow et al., 2019). In the current study, only the resulting R1 maps were used.

#### 2.5.2 MRI co-registration and intensity-scaling

The following details the additional co-registration steps conducted to create multi-sequence ensemble masks (described in 2.6.2), as well as the intensity scaling of R1 maps to reduce domain shift before segmentation with MindGlide (Goebl et al., 2025), which was trained on T1w/FLAIR-like contrasts. All registrations were performed in subject space with the T1w image as reference (0.8 mm isotropic). Volumes were reoriented to the scanner-standard orientation (using FSL’s fslreorient2std function). T1w brain masks derived from DBSegment (Baniasadi et al., 2023) were used for masking and metric computation as detailed below; transforms were always estimated on the full images unless otherwise stated. Brain masks were visually examined to confirm appropriate skull-stripping.

**R1.** We evaluated two FSL-FLIRT (Jenkinson et al., 2002) and one ANTs (Tustison et al., 2021) co-registration strategies per participant to limit the number of excluded participants and ensure acceptable alignment of R1 to T1w images. With FSL-FLIRT, we applied either (1) an initial 6-DOF rigid alignment using mutual information (spline interpolation) followed by a small affine refinement initialized from the rigid matrix (no global search; mutual information), or (2) a 6-DOF rigid alignment using normalized correlation with a T1w brain-mask weight image (spline interpolation). With ANTs, we used rigid registration (identity initialization) with default multi-resolution settings (linear interpolation). All solutions were resampled to the exact T1w grid. Where needed, the T1w brain mask was propagated into the registered R1 space (nearest-neighbor) for subsequent steps. For intensity scaling, within the T1w brain mask, R1 intensities were clipped at the 99th percentile and linearly rescaled to a 0–1000 range (voxels outside the mask were set to 0). This monotonic mapping harmonizes the dynamic range with T1w data while preserving rank order and avoiding histogram matching artifacts.

**T2-FLAIR.** FLAIR images were rigidly aligned to T1w using FLIRT (6-DOF; normalized correlation; trilinear interpolation) and resampled to the T1w grid. No intensity remapping was applied to FLAIR.

**Quality Control.** R1 and FLAIR alignment to T1w space were evaluated using (i) voxel-wise Mutual Information (MI) between T1w and registered images as an alignment metric, and (ii) visually by generating overlays in three evenly spaced slices per plane and reviewing them blinded to the metric values. Registrations were flagged as failed if MI fell below empirically determined, method-specific cut-offs (derived from pilot distributions; ≈0.25 for FSL, ≈0.30 for ANTs). For R1, when both FSL strategies passed, the higher-MI solution was retained; otherwise, ANTs was selected if it passed. If all three failed, the R1 volume was excluded. Any gross mis-registration on visual review triggered re-registration or exclusion regardless of metrics. For R1, this procedure led to the exclusion of 4 participants due to all three registration strategies failing, and the use of FSL for all except 14 participants, where ANTS produced improved registration. All FLAIR registrations passed metric thresholds and visual review.

### 2.6 MRI segmentation

#### 2.6.1 Manual GT segmentation of the thalamus

**Fig. 1:**
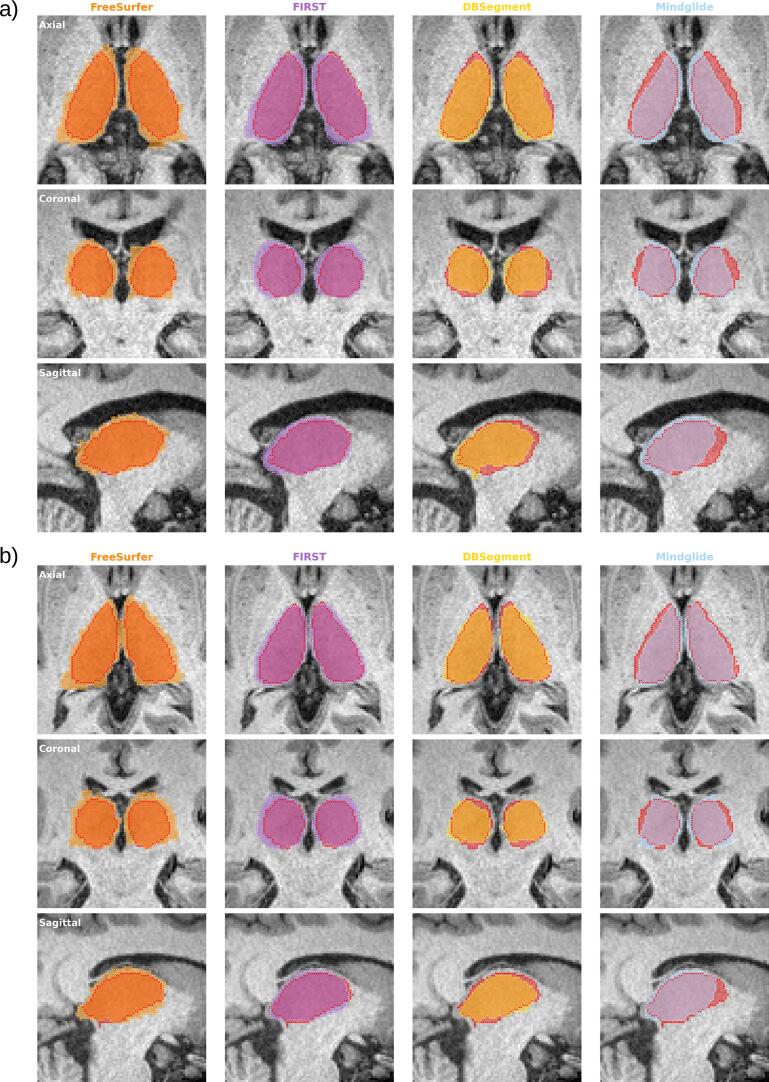
Visual comparison of FreeSurfer (orange), FIRST (purple), DBSegment (yellow) and Mindglide T1w (blue) thalamus masks overlayed on GT labels from manual segmentation (in red) from **a)** a participant with high disease burden (PMS, disease duration: 26.7 years, EDSS: 5.5, age at scan: 55) and **b)** a participant with lower disease burden (RRMS, disease duration: 1 year, EDSS: 1, age at scan: 49). (For interpretation of the references to colour in this figure legend, the reader is referred to the web version of this article.)

Manual thalamic segmentations (n = 50, see section 2.2.1) were performed by an independent rater and subsequently reviewed and, where necessary, corrected by two experts with over 15 years’ experience in MS imaging research. All delineations were conducted using ITKSnap software (Version: 4.2.0-alpha.3, available at https://www.itksnap.org) under blinded conditions. No intensity thresholding or other semi-automated tools were applied. Since there are no consensus criteria for thalamic segmentation available, the following protocol was established: using T2-FLAIR and T1w images, slice-by-slice delineation was first performed in the axial plane. Here, anatomical landmarks were the third ventricle medially, the lateral ventricle dorsally/superiorly, and the posterior limb of the internal capsule ventrolaterally (see Fig. 1). To reduce partial volume effects, ventricular mask boundaries were adjusted to visually exclude all voxels containing CSF. Laterally, where contrast is the lowest due to termination of fibers in the posterior limb of the capsula interna, the boundary was placed conservatively to avoid inclusion of adjacent white matter (Bisecco et al., 2015). After axial delineation, masks were reviewed in the coronal plane and lastly, the reconstructed 3D model was inspected for major anomalies.

#### 2.6.2 Automated thalamic segmentation approaches

Automatic segmentation of the thalamus was done using four different algorithms: two atlas-constrained algorithms, FreeSurfer (Fischl et al., 2002) and FIRST (Patenaude et al., 2011), and two deep learning-based (nnU-net) algorithms, DBSegment (Baniasadi et al., 2023), and MindGlide (Goebl et al., 2025).

**FreeSurfer (atlas-constrained)** (Fischl et al., 2002)**.** We ran FreeSurfer v7.4.1 (recon-all −all) on combined native T1w and T2w images. The thalamus (proper) labels were taken from the aseg segmentation (left/right labels 10/49), not the thalamic nuclei module. FreeSurfer estimates subcortical labels via a Bayesian atlas with subject-specific intensity modeling and topology constraints.

**FSL FIRST (atlas/shape model)** (Patenaude et al., 2011)**.** FIRST (FSL v.6.0.7; run_first_all) was applied to skull-stripped T1w images. We used a single structure list to restrict computation to the thalamus (−s L_Thal, R_Thal). We merged left/right masks into a single binary thalamus mask. In our implementation, skull stripping was performed by masking the native T1w with the corresponding DBSegment brain mask.

**DBSegment (deep learning, nnU-Net)** (Baniasadi et al., 2023)**.** We used the public DBSegment model v0.2.2 with the authors’ nnU-Net inference script (5-fold ensembling + test-time augmentation) on T1w images. DBSegment is a nnU-Net-based algorithm trained on 611 scans (from both healthy participants and patients with neurological/psychiatric disorders and clinical participants) for deep-brain structure segmentation with extensive augmentation for pathology-robustness; inference resamples to a canonical spacing and z-normalizes intensities. We retained and merged all thalamus-related labels (labels 24, 25, 26, 27, 30, and 31) from the DBSegment output.

**MindGlide (deep learning, nnU-Net)** (Goebl et al., 2025)**.** We ran MindGlide v1.0 on T1w, FLAIR, and R1 images separately. MindGlide is a nnU-Net-based algorithm trained on approximately 23,000 MS scans. For each modality, we extracted MindGlide’s DGM label, which combines the thalamus proper, accumbens area, amygdala, basal forebrain, caudate, pallidum and putamen. Since thalamus-only labels are not provided, we attempted to isolate the thalamus from the DGM label; however, the caudate and thalamus frequently formed continuous label bridges within MindGlide’s DGM output (especially at the superior thalamic boundary in coronal slices), preventing robust separation by thresholding, connected components, or morphological operations. Thalamus masks were therefore obtained by intersecting the DGM label with the subject’s FIRST thalamus mask. FIRST thalamus masks were selected because they masked out caudate voxels while consistently extending beyond the thalamus boundaries outlined by MindGlide’s DGM masks compared to other evaluated segmentation methods. The resulting thalamus masks were visually checked to confirm successful removal of caudate bridges and the absence of gross thalamic truncation (see Limitations for caveats).

**Ensemble Labels.** We generated multimodal ensemble thalamus masks by voxel-wise majority voting across the candidate binary masks (T1, FLAIR, and R1), generating three 2-sequence labels (T1-FLAIR, T1-R1, and R1-FLAIR), and one 3-sequence label (T1-FLAIR-R1). All input masks were already registered to the T1w reference. For each voxel, we computed the mean of the aligned binary masks and binarized them at a pre-specified threshold to obtain the fused label. We used a majority threshold of ≥0.5, leading to a union label for 2-sequence ensembles, and a majority label for the 3-sequence ensemble.

#### 2.6.3 Volumetric Feature Extraction

Thalamus volume was computed as the number of voxels within each thalamus mask multiplied by voxel volume (derived from the image header) and converted to mL (1 mL = 1000 mm^3^). Total intracranial volume (ICV) was extracted from the FreeSurfer output (Estimated Total Intracranial Volume; eTIV) and z-standardized by subtracting the sample ICV mean and dividing by the sample standard deviation (SD) before inclusion as a covariate in all clinical models. Longitudinal change in thalamus volume (ΔThal) for each different segmentation method was obtained per participant by computing thalamus volume at 1-year follow-up minus thalamus volume at baseline.

#### 2.6.4 Conventional assessment of percentage brain volume change

Percentage brain volume change (PBVC) between baseline and 1-year follow-up was assessed on MPRAGE images using FSL SIENA, which estimates brain atrophy by measuring the displacement of brain edge voxels between two longitudinal scans (Smith et al., 2002).

### 2.7 Statistical analysis

#### 2.7.1 Software and general statistics

All statistics and plots were conducted in Python 3.11 (pandas 1.5.3, numpy 1.24.3), with statsmodels 0.14.1 for regression models and multiple testing, Pingouin 0.5.5 for reliability metrics, SciPy 1.12 for Pearson correlations and Wilcoxon tests, as well as seaborn 0.13.2 and matplotlib 3.8.3 for plotting. Descriptive statistics for clinical variables are presented as mean ± SD, or median [interquartile range; IQR] for ordinal variables, or where distributions were non-normal based on visual inspection of residual Quantile-Quantile (QQ) plots); segmentation performance metrics are summarized as mean or median with 95% Confidence Intervals (CI) where noted. Pairwise Wilcoxon tests were used to assess statistical differences of performance measures and SIENA-derived PBVC between segmentation approaches. All multiple comparisons were controlled by adjusting p-values with the Benjamini-Hochberg method.

#### 2.7.2 Voxel-wise agreement

To assess spatial agreement with manual GT, for the 50 subjects with existing GT labels, we computed Dice-similarity coefficients (DSC), Precision, and Sensitivity from voxel-wise True Positive, False Positive, and False Negative counts, as measures of overlap between the voxels of the manually segmented and automatically generated thalamus masks (see SM.B for equations). Additionally, to characterize inter-method segmentation behavior for the entire cross-sectional dataset, we computed directed containment coefficients across every pair of T1w-derived thalamus masks generated by each segmentation algorithm, as a descriptive measure of overlap between methods. Containment coefficients are mathematically equivalent to Sensitivity, though Sensitivity is used in the context of comparison to GT. Coefficients were calculated per subject and then averaged across subjects.

#### 2.7.3 Volumetric agreement

To assess volumetric agreement between each segmentation algorithm and manual ground-truth labels, we first computed Lin’s Concordance Correlation Coefficient (CCC; (Lin, 1989), ρ_c_, which simultaneously accounts for correlation while penalizing systematic bias. CCC was calculated pairwise (GT vs algorithm) with Equation 4, where S_xy_ is the sample covariance, S_x_^2^ and S_y_^2^ the sample variances, and $\overset{¯}{x}$ and $\overset{¯}{y}$ the sample means of GT and pipeline volumes, respectively. Confidence intervals were computed via a Fisher-z transformation with asymptotic variance as described in Lin (Lin, 1989). A CCC value of ρ_c_ = 1 indicates perfect concordance, whereas 0 indicates no agreement beyond chance. For each algorithm, we additionally fit an Ordinary Least Squares (OLS; from Python statsmodels’ OLS function) model on paired subjects with algorithm-derived thalamus volume as outcome and GT volume as predictor. We report the fitted slope β with 95% CI as a dynamic range indicator (where β = 1 indicates an ideally preserved dynamic range relative to GT), and the residual standard deviation SD_e_ in mL as a precision metric.

(1) $$\rho_{c}=\frac{2S_{\mathrm{xy}}}{S_{x}^{2}+S_{y}^{2}+{\left(\overset{¯}{x}-\overset{¯}{y}\right)}^{2}}$$

To quantify absolute volumetric reliability, a single-measure ICC(3,1) (two-way mixed-effects, absolute agreement) was computed, treating GT and one fixed algorithm as the two raters in turn (Shrout and Fleiss, 1979, Koo and Li, 2016). ICC is more sensitive than CCC to within-subject scatter; large subject-specific disagreements inflate the error variance and reduce reliability even when overall bias is small. ICCs and 95% CIs were generated with Pingouin 0.5 (intraclass_corr, type = 'ICC3′).

Finally, we visualized each method’s bias and agreement with Bland-Altman plots. Volume differences between each segmentation algorithm and GT were plotted against the mean of the two volumes for each subject. We report mean bias (mean difference between methods) with SD and the 95% Limits of Agreement (LoA = mean bias ± 1.96*SD). Additionally, to assess proportional bias, we regressed the differences on the means and recorded the regression slope β with 95% CI, with larger slopes indicating higher proportional bias.

#### 2.7.4 Cross-sectional clinical associations

For the cross-sectional analysis, mass-univariate associations were conducted for each clinical variable and segmentation method. Linear regression was conducted using OLS for all variables, with clinical variables as outcomes and thalamus volumes as predictors. The effects of age at visit (in years), biological sex, and z-standardized ICV were controlled for as covariate terms. Effect size was computed as regression slope β; R^2^ values are additionally reported in Table S7 (see SM.D).

#### 2.7.5 Longitudinal clinical associations

For the longitudinal analysis, deltas for both thalamus volume and clinical outcomes were computed as the difference between 1-year follow-up and baseline (equations described in 2.3 and 2.6.3, respectively). OLS models were fitted on the clinical delta as outcome and thalamus volume delta as predictor. Age at visit, biological sex, z-standardized ICV, relapse-associated corticosteroid treatment in between sessions, and time between the two timepoints were controlled for as covariates. Only patients with available data across both timepoints for each tested clinical variable and MRI sequences were included in the analyses (see section 2.2.2). Effect size was computed as regression slope β; R^2^ values are additionally reported in Table S10 (see SM.D).

## 3 Results

### 3.1 Distribution of clinical characteristics and thalamus volume in the GT and FAS population at baseline

The 50 MS participants selected for GT segmentation had a mean age of 48.4 (± 13.0) and disease duration of 16.70 (± 12.95) years at study baseline. Their median EDSS score was 3.00 [IQR 1.50–4.00]. The mean GT label-derived thalamic volume corresponded to 9.13 (± 1.89 mL) with higher absolute mean volume in RRMS (9.44 ± 1.92 mL) compared to PMS (8.47 ± 1.70 mL).

The mean age in the cross-sectional FAS population (*n* = 321) was 43.4 ± 12.4 years, the mean disease duration was 9.19 ± 8.90 years, and the median EDSS score was 2.00 [IQR 1.50–3.00]. The cohort included patients with RRMS (*n* = 203), PMS (*n* = 16), NMOSD (*n* = 42), MOGAD (*n* = 30) and isolated syndromes including RIS, CIS and iON (*n* = 20), as well as HC (n = 10). In the FAS population for longitudinal analysis (*n* = 234), at baseline, the mean age in this population was 43.7 ± 12.0 years, the mean disease duration was 9.19 ± 8.90 years, and the median EDSS score was 2.00 [IQR 1.50–3.00]. The time between baseline visit and follow-up visit was approximately one year (.96 ± .17 years). The cohort included patients with RRMS (*n* = 162), PMS (*n* = 14), NMOSD (*n* = 24), MOGAD (*n* = 13) and isolated syndromes (*n* = 14), as well as HC (n = 7). In both FAS populations, approximately two-thirds of the participants were female (cross-sectional: *n* = 217, longitudinal: *n* = 158). As diagnostic categories were generally imbalanced in sample size, we did not conduct between-group comparisons of baseline variables. Baseline demographics, clinical characteristics, including disease stratification, are detailed in Tables S1 to S3, and Fig. S2 illustrates algorithm-derived thalamic volume distributions and means from the GT and the FAS populations (see SM.A).

### 3.2 Spatial agreement with GT

#### 3.2.1 Visual inspection of GT and algorithm-derived thalamus labels

In visual inspection, as exemplified in Fig. 1, atlas-based methods (FreeSurfer, FIRST) tended to produce larger thalamic masks than the GT, often extending into the ventricles, with FreeSurfer showing the most irregular and overextended segmentations, particularly in severely affected MS patients (Fig. 1a). FIRST yielded more rounded segmentations but still showed partial ventricular overlap. In contrast, both 3D-CNN approaches generated smaller masks than the GT but delineated the ventricular boundary more accurately; MindGlide appeared more conservative, especially along the internal capsule.

#### 3.2.2 Dice-similarity coefficient, sensitivity and precision versus GT

**Fig. 2:**
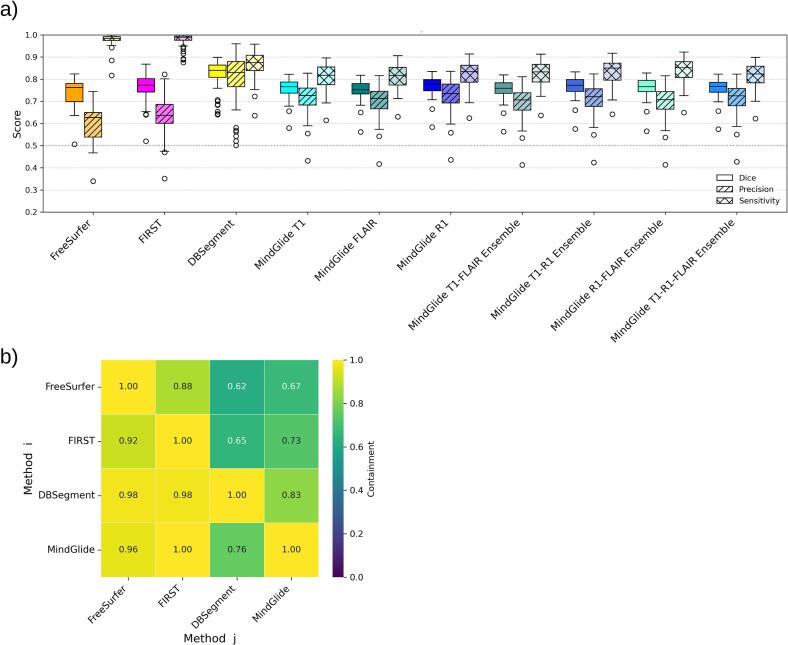
Quantification of voxel-wise agreement with GT across segmentation approaches (MS patients, *n* = 50). **a)** Tukey boxplots of DSC, precision, and sensitivity measures for each approach computed voxel-wise against the manual GT. Only descriptive statistics are shown: boxes indicate the IQR (Q1–Q3), the central line is the median, and whiskers extend to 1.5 × IQR from the quartiles. **b)** Containment coefficients obtained from T1w MRI segmentations. The heatmap illustrates the degree to which method i is contained within method j. Lighter (higher) values in the rows indicate a larger degree of containment, while lighter values in the columns indicate that the corresponding labels are larger, and therefore, contain the other labels. Lighter rows and darker columns for a method indicate under-segmentation, while darker rows but lighter columns indicate over-segmentation.

To quantify spatial segmentation performance relative to the manual GT, we compared Dice-similarity coefficients (DSC), sensitivity, and precision across all methods; Fig. 2a and Table 1 summarize the medians and IQR across methods. Pairwise comparison tables are additionally included in Table S4 (see SM.B). In relation to GT, DBSegment achieved a DSC of 0.840, significantly outperforming all other methods (p < 0.001). MindGlide R1 masks had the second highest DSC with 0.777, which differed significantly from all labels (p < 0.002 to 0.001) except FIRST masks (DSC = 0.773, p = 0.466). Similarly, MindGlide T1 masks achieved a DSC of 0.766, which significantly differed from other compared labels (p < 0.001) except FIRST (p = 0.396), MindGlide T1-R1-FLAIR ensembles (DSC = 0.767, p = 0.805) and FreeSurfer (DSC = 0.762, p = 0.055). As detailed in Table 1, MindGlide FLAIR masks had the lowest DSC (0.752), but did not significantly differ from FIRST (p = 0.055) and FreeSurfer (p = 0.362).

**Table 1:** Overview of DSC, Precision, and Sensitivity medians and Interquartile Ranges (IQR) across algorithm-derived thalamus segmentations in relation to GT.

Segmentation method	Dice median (IQR)	Precision median (IQR)	Sensitivity median (IQR)
Freesurfer	0.762 (0.698–0.781)	0.627 (0.538–0.649)	0.984 (0.975–0.992)
FIRST	0.773 (0.742–0.802)	0.637 (0.601–0.686)	0.989 (0.975–0.994)
DBSegment	0.840 (0.808–0.864)	0.829 (0.766–0.880)	0.874 (0.839–0.908)
MindGlide T1	0.766 (0.737–0.787)	0.727 (0.683–0.760)	0.817 (0.775–0.855)
MindGlide FLAIR	0.752 (0.734–0.780)	0.713 (0.666–0.745)	0.816 (0.773–0.853)
MindGlide R1	0.777 (0.748–0.798)	0.735 (0.693–0.778)	0.834 (0.787–0.862)
MindGlide T1-FLAIR Ensemble	0.759 (0.736–0.784)	0.706 (0.660–0.739)	0.833 (0.787–0.867)
MindGlide T1-R1 Ensemble	0.772 (0.745–0.798)	0.720 (0.677–0.756)	0.850 (0.796–0.872)
MindGlide R1-FLAIR Ensemble	0.765 (0.744–0.794)	0.707 (0.665–0.744)	0.851 (0.807–0.879)
MindGlide T1-R1-FLAIR Ensemble	0.767 (0.741–0.785)	0.725 (0.680–0.758)	0.824 (0.783–0.859)

For precision, DBSegment masks outperformed all other methods (p < 0.001) with a precision median of 0.829, indicating a comparatively lower proportion of false positives among voxels labeled as thalamus (Fig. 2a). Freesurfer performed the worst, followed by FIRST, with precision medians of 0.627 and 0.637, respectively (p < 0.001). All MindGlide masks had intermediate precision, ranging from 0.735 at the higher end (for the R1-derived masks) and 0.706 at the lower end (for the T1-FLAIR ensemble). All precision values significantly differed from each other (p < 0.001) (also see Table S4, SM.B).

In contrast, for sensitivity, FIRST and FreeSurfer masks significantly outperformed all other segmentation methods (p < 0.001), with sensitivity medians of 0.989 and 0.984, respectively, indicating a comparatively higher rate of true positives (and fewer missed thalamus voxels). DBSegment showed the third highest sensitivity with 0.874, which significantly differed from every other method (p < 0.001). MindGlide FLAIR and T1 masks performed the worst, with sensitivity medians of 0.816 and 0.817, respectively, significantly lower than all (p < 0.03 to 0.001) other masks except each other (p = 0.977). The rest of the MindGlide masks obtained intermediate sensitivity medians, ranging from 0.851 at the higher end (for the R1-FLAIR ensemble) to 0.824 at the lower end (for the T1-R1-FLAIR ensemble), all differing significantly from each other (p < 0.001) except the R1 masks and T1-FLAIR ensemble (p = 0.914).

#### 3.2.3 Containment coefficients

To assess spatial overlap and inter-method segmentation behavior in the cross-sectional FAS population (*n* = 321 participants), we generated a containment coefficient heatmap based on all available T1w thalamus masks derived from the four tested segmentation algorithms. The results provide a descriptive overview of relative mask extent and overlap between methods and do not, by themselves, establish segmentation quality in the absence of GT. Instead, the heatmap helps characterize whether a method tends to behave as a conservative subset or a liberal superset of others. As shown in Fig. 2b, DBSegment yielded the most conservative (smallest) labels, with high containment in all other methods but only partial reciprocal coverage, indicating under-segmentation. FIRST and FreeSurfer showed the opposite pattern, with relatively larger segmentations that contained other methods well but were not fully contained within themselves, indicating over-segmentation. Comparatively, MindGlide T1 demonstrated the most balanced profiles, with both moderate containment and coverage.

### 3.3 Volumetric agreement with GT

**Fig. 3:**
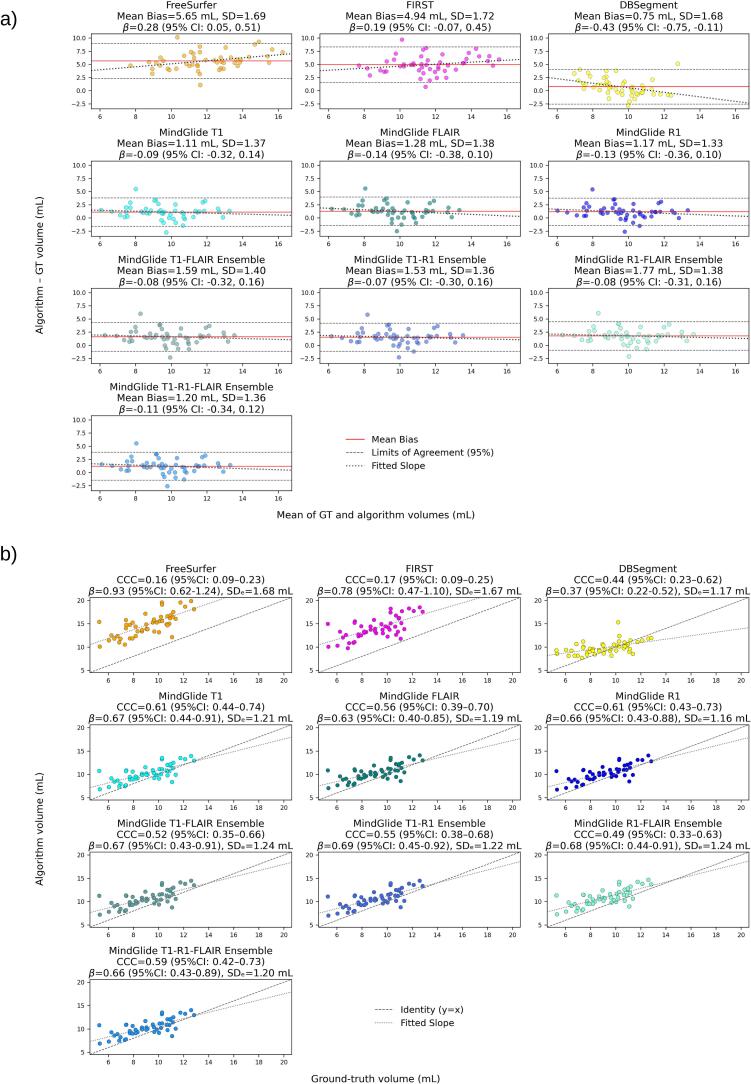
Quantification of volumetric agreement with GT across segmentation approaches (MS patients, *n* = 50). **a)** Scatter plots of algorithm vs GT thalamus volumes, annotated with Lin’s CCC and 95% CI, and regression slope β with 95% CI and standard deviation of residuals (SD_e_); **b)** Bland-Altman plots with reported mean bias and SD, as well as regression slope β for error vs bias with 95% CI.

To complement voxel-wise overlap and inter-method containment, we additionally quantified volumetric agreement with GT to capture systematic over-/under-segmentation and absolute reliability that spatial overlap alone cannot account for. Fig. 3a shows scatterplots of algorithm-derived versus GT thalamus volume (n = 50), annotated with CCC values with 95% CI, and the algorithm-GT regression slopes β with 95% CI and residual SD (SD_e_; also see Table S5, SM.C). MindGlide outperformed other segmentation algorithms in concordance with GT, with T1 and R1 masks having the highest CCC (T1: ρ_c_ = 0.61, 95% CI [.44, 0.74]; R1: ρ_c_ = 0.61, 95% CI [.43, 0.73]) followed closely by the T1-R1-FLAIR ensemble (ρ_c_ = 0.59, 95% CI [.42, 0.73]). MindGlide slopes were consistent across variants (β = 0.63 to 0.69) and indicated mild but uniform range compression. DBSegment ranked second by CCC (ρ_c_ = 0.44, 95% CI [.23−0.62]) but had the lowest slope (β = 0.37, 95% CI [.22, 0.52]), consistent with highly compressed dynamic range (i.e., increasing underestimation at larger GT volumes). FreeSurfer and FIRST had the lowest concordance with GT (ρ_c_ = 0.16, 95% CI [.09, 0.23] and ρ_c_ = 0.17, 95% CI [.09, 0.25] respectively), but the largest slopes (β = 0.93, 95% CI [0.62, 1.24] and 0.78, 95% CI [0.54, 1.10] respectively), indicating large systematic offsets despite preserved dynamic ranges relative to GT.

As detailed in Table 2, MindGlide also outperformed other methods in terms of ICC(3,1), with the R1-derived masks and the T1-R1 ensembles achieving the highest agreement of individual algorithm-derived volumes with GT (ICC = 0.731 (95% CI [.57, 0.84] for both), followed closely by its T1 masks (ICC = 0.72, 95% CI [.56, 0.83]). In contrast to CCC ranking, FreeSurfer masks had the second highest agreement (ICC = 0.70, 95% CI [.53, 0.82]) followed by FIRST (ICC = 0.66, 95% CI [.47, 0.79]). DBSegment exhibited the lowest agreement (ICC = 0.49, 95% CI [.24, 0.68], indicating large within-subject disagreement relative to GT despite a moderate CCC, but in line with the high dynamic range compression suggested by its slope.

**Table 2:** Single-measure Intra-Class Correlation (ICC(3,1)) with 95% CI for Ground-Truth vs Segmentation Method.

Method	ICC(3,1)	CI95%
Freesurfer	0.705	[0.53, 0.82]
FIRST	0.659	[0.47, 0.79]
DBSegment	0.489	[0.24, 0.68]
MindGlide T1	0.725	[0.56, 0.83]
MindGlide FLAIR	0.706	[0.53, 0.82]
MindGlide R1	0.731	[0.57, 0.84]
MindGlide T1-FLAIR Ensemble	0.715	[0.54, 0.83]
MindGlide T1-R1 Ensemble	0.731	[0.57, 0.84]
MindGlide R1-FLAIR Ensemble	0.721	[0.55, 0.83]
MindGlide T1-R1-FLAIR Ensemble	0.722	[0.56, 0.83]

As illustrated in Fig. 3b, we additionally performed Bland-Altman bias analyses (see supplementary Table S6 for full tabularized results with slope and LoA values, SM.C). While DBSegment revealed the lowest overall mean bias (.75 ± 1.68 mL), it showed a large negative proportional bias (with β = −0.43, 95% CI [−0.75, −0.11], or a loss of 0.43 mL for each additional 1 mL of true thalamus volume), indicating a tendency to overestimate smaller but underestimate bigger thalamus volumes. MindGlide had the second lowest mean bias, especially for its T1 (mean bias = 1.11 ± 1.37 mL) and R1 masks (mean bias = 1.17 ± 1.33 mL), and had the lowest proportional bias across sequences and ensembles compared to other methods (e.g., β = −0.09, 95% CI [-0.32, 0.14] for T1; β = −0.14, 95% CI [−0.36, 0.10] for R1). FreeSurfer showed the worst overall performance, with the highest overall mean bias (5.65 ± 1.69 mL) and relatively high positive proportional bias (β = 0.28, 95% CI [.05, 0.51], or a gain of 0.28 mL per additional 1 mL of true thalamus volume), indicating a tendency to underestimate smaller volumes and overestimate bigger volumes. FIRST had the second-highest mean bias (4.94 ± 1.72 mL), but showed relatively smaller proportional bias (β = 0.19, 95% CI [-0.07, 0.45]) compared to DBSegment and FreeSurfer (Fig. 3b; Table S6 in SM.C).

### 3.4 Cross-sectional analysis of thalamus volume in relation to clinical outcomes

We focused our subsequent clinical outcome analyses on FIRST as the best-performing atlas-based method and on the nnU-net algorithms; FreeSurfer was omitted given its lower performance and higher proportional bias compared to FIRST in GT agreement analyses (see Limitations for further discussion). To investigate and compare the clinical relevance of the remaining segmentation algorithms’ thalamus masks in the FAS cohort (*n* = 321), we first performed a cross-sectional analysis at study baseline to assess associations of extracted absolute thalamus volumes with EDSS score, T25FW, 9HPT, and SDMT score, as well as disease duration. Linear regression (OLS) models were fitted for each segmentation-derived volume, and model performance was evaluated. Complete model results are available in Table S7 (SM.D).

Based on visual inspection of residual QQ plots, clinical outcomes were normally distributed except T25FW and 9HPT, which were right-skewed and were therefore log-transformed before entering the OLS model, yielding an acceptable distribution. Homoscedasticity assumptions were assessed with visual inspection of Residual vs Fitted plots. EDSS and disease duration (years) showed heteroscedasticity; an OLS with robust standard errors (HC3) was therefore fitted for both.

**Fig. 4:**
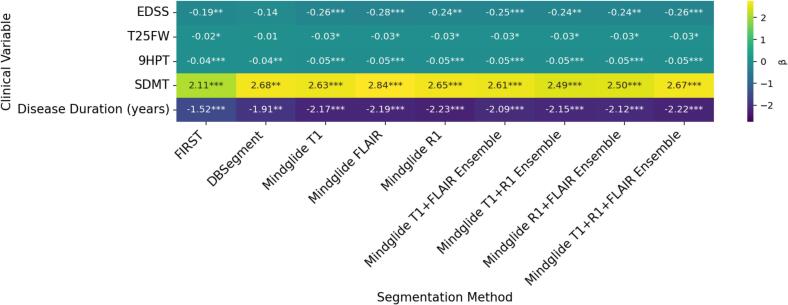
Summary of cross-sectional analysis OLS results (*n* = 321 participants in the cross-sectional FAS). Due to missing clinical outcome values, sample sizes differ slightly across outcomes, with n = 309 for EDSS score, n = 310 for T25FW, n = 315 for 9HPT, n = 312 for SDMT score, and n = 310 for disease duration. Effect sizes reported are regression coefficients β, representing change per unit of clinical outcome (or percent change for log-transformed variables T25FW and 9HPT) for every 1 mL increase in thalamic volume. OLS models were adjusted for age, sex, and standardized ICV. EDSS and disease duration (years) were fitted with robust standard errors (HC3) due to heteroscedasticity. Asterisks indicate statistical significance, with * for p < 0.05, ** for p < 0.01, and *** for p < 0.001.

As shown in Fig. 4, across most clinical outcomes, MindGlide thalamus volumes generally outperformed FIRST’s and DBSegment’s in terms of statistical significance and effect size β. For instance, MindGlide T1 volumes showed a highly significant negative association between EDSS and thalamus volume (β = −0.26, SE = 0.07, p < 0.001), indicating a 0.26 decrease in EDSS score for each mL increase in thalamus volume. FIRST volumes showed a relatively weaker association with EDSS (β = −0.19, SE = 0.06, p = 0.002). In general, DBSegment volumes did not show any statistically significant association with EDSS (β = −0.14, SE = 0.11, p = 0.221) or T25FW (β = −0.01, SE = 0.02, p = 0.672). In contrast, FIRST and MindGlide volumes showed weak negative associations with T25FW (respectively, β = −0.02, SE = 0.01, p = 0.016, and β = −0.03, SE = 0.01, p = 0.014 for T1). Nevertheless, DBSegment volumes showed a stronger positive association with SDMT (β = 2.68, SE = 0.94, p = 0.006) compared to FIRST (β = 2.12, SE = 0.48, p < 0.001) and most MindGlide variants (e.g., β = 2.63, SE = 0.63, p < 0.001 for T1), though the association was stronger for MindGlide FLAIR volumes (β = 2.84, SE = 0.65, p < 0.001). DBSegment volumes also showed a relatively stronger negative association with disease duration (β = −1.91, SE = 0.67, p = 0.006) compared to FIRST’s (β = −1.52, SE = 0.38, p < 0.001), though MindGlide’s outperformed both (e.g., β = −2.17, SE = 0.49, p < 0.001 for T1). In general, MindGlide variants did not yield marked differences in predictive power across tested outcomes compared to the MindGlide T1 volumes (also see Table S7, SM.D).

Post-hoc sensitivity analyses restricted to the RRMS cohort yielded broadly consistent cross-sectional association patterns (see SM.D II, Table S11 and Fig. S3), with two notable differences for DBSegment thalamus volumes: slightly stronger associations with EDSS (β = −0.29, SE = 0.09, p = 0.002) compared to other methods, and a statistically significant association with T25FW (β = −0.03, SE = 0.02, p = 0.042).

### 3.5 Longitudinal analysis of thalamic volume change

**Fig. 5:**
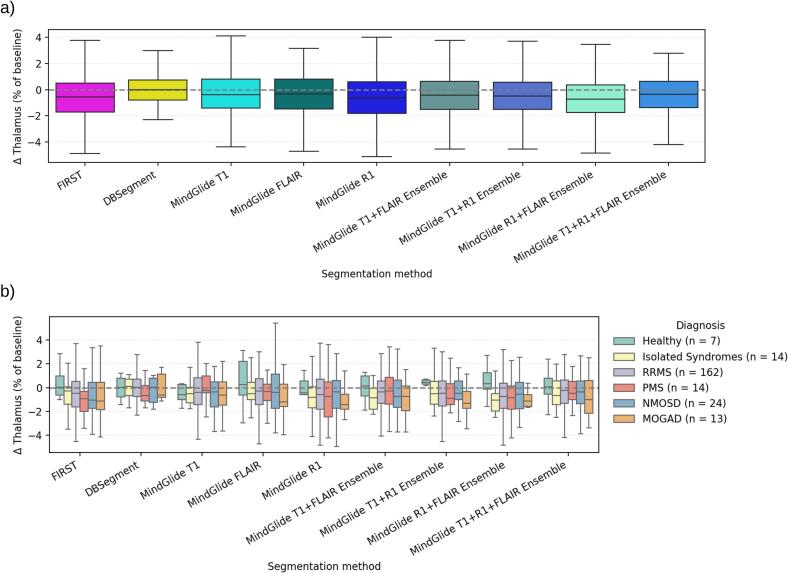
Quantification of thalamic volume change (in % of baseline) between baseline and 1-year follow-up, captured by the different segmentation algorithms, is shown for **a)** all participants across the entire longitudinal FAS cohort *(n* = 234), and **b)** stratified by diagnosis. Only descriptive statistics are shown: boxes indicate the IQR (Q1–Q3), the central line is the median, and whiskers extend to 1.5 × IQR from the quartiles.

We further performed a longitudinal analysis of relative thalamic volume change from baseline to 1-year follow-up captured by the algorithm-based segmentation methods (excluding FreeSurfer) in the longitudinal FAS cohort (*n* = 234). As illustrated in Fig. 5a, the highest average thalamic volume loss was generally detected by the MindGlide R1-FLAIR ensemble (Δ-Thal = –0.70 ± 2.03%) and MindGlide R1 (–0.65 ± 2.16%), while DBSegment captured the lowest volume loss (–0.06 ± 1.55%; all p < 0.02 vs. other methods). FIRST detected a mean delta of –0.63 ± 1.55%, significantly higher than most methods (all p < 0.009) except MindGlide R1 (p = 0.236) and the R1-FLAIR ensemble (p = 0.381). Among MindGlide variants, T1 segmentations yielded the lowest delta (Δ-Thal = –0.34 ± 2.11%), significantly lower than most methods (p < 0.030) except FLAIR (p = 0.270), T1-R1 (p = 0.177), and T1-FLAIR-R1 ensembles (p = 0.219). MindGlide R1 segmentations showed a higher delta than DBSegment (p < 0.001), MindGlide T1 (p = 0.026), and T1-FLAIR-R1 ensembles (p = 0.020), but did not differ from FIRST (p = 0.236). Full delta analysis results, including absolute mean values in mm^3^, are detailed in Table S8, and full pairwise statistics are provided in Table S9 (see SM.D).

As shown in Fig. 5b, additional stratification of captured Δ-Thal (%) by diagnosis revealed broadly similar patterns across methods (see Table S8 for stratified delta descriptive statistics, SM.D). In healthy controls (n = 7), distributions were centered near zero with small, often slightly positive medians. Across patient groups, most methods showed negative medians (volume loss); DBSegment was the exception, with means closer to zero, non-negative in NMOSD, and generally the lowest variability (narrowest IQR/whiskers). In MS brains (RRMS n = 162; isolated syndromes including RIS, CIS and iON, n = 14; PMS n = 14), MindGlide-based labels captured broadly comparable thalamus volume declines across groups, with CIS and PMS showing somewhat more atrophy compared to RRMS, albeit inconsistently. Using FIRST and DBSegment, PMS exhibited the largest median volume loss. Furthermore, NMOSD (n = 24) showed intermediate changes similar to RRMS, whereas MOGAD (n = 13) displayed the most pronounced negative shifts across most MindGlide variants. Owing to unequal and small group sizes, these observations are reported descriptively without formal between-group testing (Fig. 5b).

We also tested associations of thalamus atrophy detected per method and SIENA-derived total PBVC. We observed significant positive associations for all segmentation algorithms’ masks (see Fig. S6, SM.E). Our longitudinal cohort had a mean total PBVC of –0.32 ± 0.55%. On average, thalamus atrophy detected by MindGlide T1 and ensembles showed the strongest associations with PBVC (Pearson r = 0.52 to 0.54, p < 0.001), though pairwise comparisons showed no significant differences between methods except with DBSegment (p < 0.01 to 0.001). DBSegment-derived thalamus volume change had the weakest association with PBVC (Pearson r = 0.14, p = 0.03); significantly lower than all other methods (p < 0.01 to 001). Pairwise comparison results are additionally included in Table S16 (see SM.E).

### 3.6 Longitudinal change in clinical outcomes and association with thalamus volume loss

For analysis of longitudinal change in clinical outcome variables, we assessed the delta of selected clinical outcome variables between baseline and 1-year follow-up visit. Across the entire cohort, changes were generally small with high variability. All delta variables passed the normality assumption following a residual QQ plot inspection. EDSS and 9HPT deltas showed heteroscedasticity following a visual inspection of Residuals vs Fitted plots; an OLS with robust standard error (HC3) was therefore fitted for both.

Descriptive statistics for all clinical deltas, stratified by diagnosis, are provided in Table S8 (SM.D). On average, EDSS showed negligible changes across diagnoses, though there was a small increase in NMOSD. T25FW showed generally small changes across groups, with somewhat larger increases in PMS. 9HPT deltas were negligible across all groups. Furthermore, SDMT performance tended to improve slightly, particularly in RRMS, MOGAD, and RIS/CIS/iON groups (see Table S8, SM.D).

**Fig. 6:**
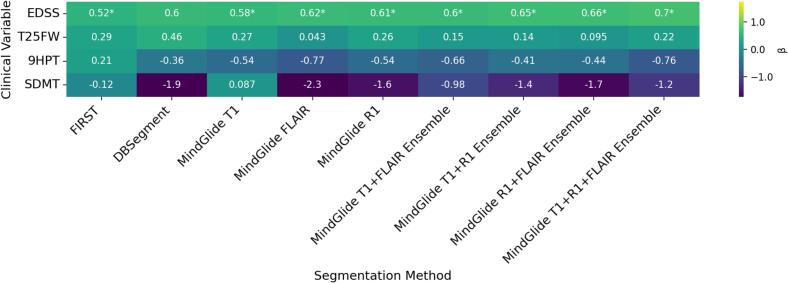
Longitudinal associations between thalamus volume change and clinical outcome change (*n* = 234 participants in the longitudinal FAS). Due to missing values for clinical outcomes, sample sizes differ slightly across clinical outcomes, with n = 221 for EDSS score, n = 219 for T25FW, n = 222 for 9HPT, and n = 220 for SDMT score. Asterisks indicate statistical significance, with * for p < 0.05, ** for p < 0.01, and *** for p < 0.001.

We further investigated whether lower thalamic atrophy rates between baseline visit and follow-up, assessed by the different segmentation algorithms, were associated with less progression in clinical outcomes. Fig. 6 summarizes the regression slope β per method, illustrating the unit change in clinical outcomes associated with 1 mL change in thalamus volume. Positive β values indicate a positive association between changes in clinical outcome scores (Δclinical) and changes in thalamus volume (ΔThal, where more negative values reflect greater atrophy and less negative or positive values indicate less volume loss). Out of the four tested clinical outcomes, only changes in EDSS showed significant associations with changes in thalamus volumes across methods (p < 0.05) after correction for multiple comparisons, except for DBSegment’s (β = 0.60, SE = 0.25, p = 0.069). Surprisingly, the direction of the association was positive across methods. The MindGlide T1-FLAIR-R1 ensemble volume yielded the highest effect size (β = 0.70, SE = 0.25, p = 0.032), indicating a 0.7 increase in EDSS score per 1 mL less thalamic volume loss. The MindGlide R1-FLAIR and T1-R1 ensemble volumes were close seconds (β = 0.66, SE = 0.23, p = 0.032 and β = 0.65, SE = 0.23, p = 0.032, respectively). Comparatively, FIRST volumes had the lowest effect size out of the significant associations with EDSS progression (β = 0.52, SE = 0.16, p = 0.032). Complete longitudinal model results are detailed in Table S10 (SM.D). Post-hoc sensitivity analyses restricted to the RRMS cohort showed a similar effect-size pattern across methods but did not retain statistically significant associations after FDR correction (see SM.D II, Table S12 and Fig. S4), consistent with reduced power in the stratified cohort.

Additional post-hoc sensitivity analyses were conducted to confirm the unexpected direction of the association (see SM.D III). The positive association between change in EDSS and change in thalamus volume was robust to winsorization of thalamus volume changes (1–99% and 5–95%, Tables S13 and S14 respectively), indicating that results were not driven by extreme longitudinal volume changes. Nevertheless, following multiple comparison corrections, associations were only weakly or marginally significant with 1–99% winsorization, and marginally or not significant with 5–95% winsorization. In addition, an ordinal logistic regression of EDSS-change categories (Table S15, Fig. S5) yielded consistent directionality across methods, supporting that the observed effects were not an artefact of treating EDSS change as continuous, though associations were only marginally significant following multiple comparison corrections.

## 4 Discussion

In this study, we benchmarked different thalamus‐segmentation methods in patients with MS, NMOSD, and MOGAD, and investigated whether adding microstructural information by including quantitative MRI improves performance and clinical relevance. To that end, we first obtained T1w thalamus masks derived from four segmentation algorithms, ranging from classical atlas-constrained (Freesurfer and FIRST) to nn-Unet (MindGlide and DBSegment) models. Second, leveraging MindGlide’s multiple-contrast capability, we generated FLAIR- and R1-based thalamus masks and computed union and majority-voted ensembles as an exploratory proof-of-concept extension. Performance was evaluated against manual GT using voxel-wise and volumetric agreement. To investigate the clinical relevance of the segmentation methods, we additionally quantified cross-sectional and ∼1-year longitudinal associations between thalamic volume (loss) and clinical outcomes in an extended patient population.

Out of the four tested algorithm-derived segmentation approaches, DBSegment achieved the highest DSC and precision, indicating the best voxel-wise agreement and the lowest false-positive rate relative to manual GT. Its sensitivity was modestly lower than that of FIRST and FreeSurfer, while containment coefficients indicated a mild tendency to under-segment compared to the other algorithms. In contrast, FIRST and FreeSurfer masks showed the lowest precision (≈24% below DBSegment), and the highest sensitivity (≈12% above DBSegment), consistent with over-segmentation relative to GT, while also exhibiting the lowest containment coefficients. MindGlide yielded intermediate precision (≈12% below DBSegment for T1w) and the lowest sensitivity (≈5% below DBSegment for T1w), though compared to atlas-constrained methods, its precision/sensitivity and containment profiles were more balanced. This pattern is consistent with prior reports that atlas-constrained pipelines such as FreeSurfer and FIRST are vulnerable to lesion- and atrophy-induced intensity distortions and misregistration in MS, particularly in DGM, leading to reduced precision and degraded containment (Gelineau-Morel et al., 2012, González-Villà et al., 2017) as well as significantly lower DSC compared to GT than HCs for the thalamus and putamen (de Sitter et al., 2020). By contrast, owing to their data-driven approach as opposed to reliance on atlas priors, CNN-based methods such as nnU-Net typically achieve higher spatial fidelity in pathological datasets (Opfer et al., 2023, Isensee et al., 2021, de Sitter et al., 2021), as well as improved inter- and intra-scanner variability and cross-sectional thalamic atrophy detection, even when trained on healthy controls (Opfer et al., 2023). Atlas-based approaches in our study consistently over-segmented MS brains, most likely due to lesion- and atrophy-related boundary ambiguities that interfere with atlas priors, whereas 3D-CNNs may partly mitigate these effects by learning data-driven features.

When evaluated for absolute volumetric agreement with GT, the performance ranking of the segmentation methods changed. All MindGlide variants, particularly its T1, R1, and T1-R1-FLAIR ensemble masks, yielded the highest CCC with GT, along with the highest single-measure volume reliability (as measured with ICC 3,1). DBSegment, despite its superior spatial agreement, achieved only moderate volumetric concordance, the lowest ICC, and a regression slope indicating proportional bias: smaller thalami tended to be over-segmented, larger ones under-segmented in our GT population of MS patients. On the other hand, atlas-constrained methods achieved higher ICCs than DBSegment, but at the cost of the largest systematic biases and lowest overall concordance with GT, despite having the most preserved dynamic range (as suggested by their slopes vs GT volumes). FreeSurfer additionally showed proportional bias in the opposite direction of DBSegment, underestimating small and overestimating larger thalamus volumes.

This aligns well with previous reports demonstrating proportional bias for atlas-constrained approaches such as FreeSurfer, where thalamic volumes in patients with MS were consistently overestimated compared to manual GT, particularly in highly atrophic thalami (Burggraaff et al., 2021). While atlas-based methods such as FIRST (Lyman et al., 2022) and FreeSurfer (Burggraaff et al., 2021) generally reveal large systematic offsets, our results indicate that they still may be acceptable for rank-ordering subjects by thalamus size, given their relatively preserved dynamic range and ICC. The fact that DBSegment showed proportional bias, whereas MindGlide achieved the most accurate volumetrics, further suggests that voxel-wise spatial overlap does not necessarily translate into volumetric accuracy. Conversely, good volumetric agreement can still arise from anatomically implausible segmentations; therefore, segmentation quality is best assessed by triangulating complementary metrics.

Given that MindGlide was trained and validated on a large MS cohort (Goebl et al., 2025), our findings also align with reports that disease-specific training improves segmentation accuracy in atrophied structures (de Sitter et al., 2020). This supports the notion that pathology-induced structural alterations (i.e., inflammatory lesions and diffuse neurodegeneration) in MS brains may fundamentally alter the intensity-volume relationship. Among the algorithm-derived methods tested, MindGlide thus appears to be the most suitable approach for absolute thalamus volume measurements and rank-ordering volumes in MS cohorts.

Relative to T1w-only input, MindGlide variants using R1 or FLAIR alone or their ensembles yielded modest improvements in MindGlide’s thalamus segmentation performance relative to GT. R1 masks and T1-R1 or T1-FLAIR-R1 ensembles slightly improved voxel-wise agreement and ICC compared to T1, although a small increase in mean bias accompanied these gains. In contrast, FLAIR, T1-FLAIR, and R1-FLAIR ensembles showed a slight reduction in volumetric agreement compared to T1. Given that qMRI maps convey biologically meaningful microstructural information (Weiskopf et al., 2013, Weiskopf et al., 2021, Weiskopf et al., 2015, Leutritz et al., 2020) of pathological relevance to MS, where neuroinflammation, demyelination, and iron accumulation shape disease-related tissue changes (Pontillo et al., 2021, Bonnier et al., 2017, Chen et al., 2025, Vavasour et al., 2022, Granziera et al., 2021, Pontillo et al., 2023), we expected R1 map input to MindGlide to improve the performance of thalamic delineation in patients with neuroinflammatory disorders. A plausible explanation for the observed marginal improvement may be explained by the acquisition parameters of the available MPM sequence (Cooper et al., 2020, Trang et al., 2024). The spatial resolution of R1 maps (1.6 mm^3^) was substantially worse than that of T1w images (0.8 mm^3^), potentially limiting sensitivity to small anatomical boundaries in DGM regions such as the thalamus and subfield delineation (Levy et al., 2022).

In our extended longitudinal cohort, MindGlide, on average, captured an annual thalamic volume decline of between 0.3–0.7% depending on the variant, similar to FIRST (0.6%), both consistent with prior studies reporting annual DGM atrophy rates of ∼0.4–1% in MS (Azevedo et al., 2018, Uher et al., 2019, Opfer et al., 2025). Meanwhile, DBSegment detected near-zero change on average, potentially reflecting the proportional bias we observed in our volumetric agreement analysis. Stratified descriptive statistics confirmed previously observed atrophy patterns across diagnostic groups: healthy controls remained stable, while thalamus atrophy occurred as early as in CIS (Kipp et al., 2015, Tommasin et al., 2021) and was mostly consistent across MS subtypes (Azevedo et al., 2018). In addition, MOGAD patients exhibited the strongest declines across most MindGlide variants, in line with prior studies showing greater thalamic atrophy in MOGAD compared to MS and NMOSD (Rechtman et al., 2022, Lotan et al., 2023). Nevertheless, thalamic atrophy in NMOSD patients remains contentious. In some AQP4-IgG-positive cohorts, DGM volumes were not found to differ from HC (Finke et al., 2016, Papadopoulou et al., 2019). This could relate to the disease’s neuropathological predilection for AQP4-rich circumventricular/periependymal regions (Kim et al., 2015) and a potential predominance of attack-related anterograde degeneration rather than primary damage to DGM nuclei (Papadopoulou et al., 2019).

In our cross-sectional analysis of clinical outcomes, thalamus volumes derived from MindGlide showed the most consistent associations with clinical outcomes of disability (EDSS, T25FW, 9HPT) and cognitive impairment (SDMT), whereas DBSegment lacked significant associations with EDSS and T25FW. For MindGlide, the inclusion of FLAIR or R1, either alone or in ensembles, did not markedly change predictive power compared to T1 masks. These observations align with extensive clinical evidence demonstrating that smaller thalamic volume correlates with worse disability (Raji et al., 2018, Jakimovski et al., 2018, Bergsland et al., 2021) (incl. EDSS, T25FW, 9HPT) or cognitive performance (incl. SDMT) in MS (Bergsland et al., 2021, Naghavi et al., 2023, Bisecco et al., 2018, Noteboom et al., 2023), and extend to MOGAD (Lotan et al., 2023), and NMOSD (Hyun et al., 2017, Seok et al., 2022).

In the longitudinal analysis relating one-year changes in thalamic volume to changes in clinical outcomes, only EDSS change showed associations with thalamic volume change across methods, whereas MSFC measures did not. Notably, the association between change in EDSS and change in thalamus volume was positive, indicating that greater EDSS worsening was associated with less thalamic volume loss (i.e., more positive volume change). This direction is counterintuitive given the extensive cross-sectional literature linking lower thalamic volume to greater disability.

Several factors may contribute to this unexpected directionality. First, EDSS is ordinal and influenced by relapse–recovery dynamics and measurement variability, such that short-interval EDSS changes do not necessarily track monotonic neurodegenerative volume loss (Kalincik et al., 2015, Meyer-Moock et al., 2014, Hirst et al., 2012). Second, longitudinal volume differences can be affected by pseudoatrophy or treatment-related changes in tissue water content and inflammation (Zivadinov et al., 2008, De Stefano et al., 2021), which may decouple apparent volume change from irreversible tissue loss. Third, longitudinal segmentation variability and scanner/session effects introduce noise in computed volume changes, and apparent increases can occur even when true change is negative (Takao et al., 2021). Consistent with this, the association weakened after winsorization and did not remain robust after multiple-comparison correction in all sensitivity analyses. Therefore, we interpret the longitudinal EDSS findings as exploratory rather than a definitive validation of longitudinal anatomical accuracy.

Notably, while neither FLAIR nor R1 input improved cross-sectional model performance over T1-based MindGlide masks, both strengthened the modest but consistent longitudinal associations between MindGlide-derived thalamus atrophy and changes in EDSS. A possible explanation is that these contrasts add progression-sensitive signal: longitudinal decreases in R1 have been reported in deep gray matter, including the thalamus, and relate to cognitive and motor disability (Pontillo et al., 2023), and consensus recommendations emphasize 3D-FLAIR as a clinical standard for MS monitoring, often sufficient without T1w or contrast enhancement (Wattjes et al., 2021). At the same time, while several segmentation pipelines support FLAIR due to their central role in lesion detection (e.g., (Fischl et al., 2002, Puonti et al., 2016), MPM-derived R1 is rarely integrated for segmentation. Although exploratory, our findings therefore indicate that incorporating R1 alongside FLAIR and T1w images may improve longitudinal tracking of disease progression in MS and related neuroinflammatory disorders, at least in relation to thalamic atrophy (Pontillo et al., 2019, Motamedgorji et al., 2025, Fuchs et al., 2021, Diaz-Hurtado et al., 2022, Zivadinov et al., 2018, Akaishi et al., 2020, Zivadinov et al., 2024).

Nevertheless, while stronger associations between volumetric measurements and clinical and cognitive measures may support the plausibility of a segmentation method, they can also be influenced by confounding factors such as disease heterogeneity, global brain atrophy, measurement noise in clinical scores, and limited sensitivity of clinical scales. Clinical associations with volumes should therefore be interpreted as supportive rather than definitive evidence of superior anatomical accuracy.

In addition, we observed a stronger positive association between FIRST and MindGlide-derived percentage thalamus volume change and SIENA-derived total PBVC compared to DBSegment. Consistent with reports that thalamic atrophy predicts whole brain atrophy in MS (Fujimori and Nakashima, 2024), our results support that FIRST, and MindGlide in particular, can extract biologically meaningful signal from thalamic change. Although stronger associations with PBVC do not necessarily indicate improved segmentation accuracy, these findings further tie into our previous discussion: the proportional bias observed in DBSegment-derived volumes may put it at a disadvantage in tracking longitudinal changes compared to atlas-constrained methods, which better preserved the dynamic range of volumes despite a strong overestimation tendency. Meanwhile, despite sharing the same underlying deep learning architecture as DBSegment, MindGlide offered the most balanced overall performance, likely due to having a larger and MS-specific training dataset.

## 5 Limitations

This study has several limitations. First, MindGlide thalamus masks were derived by intersecting MindGlide’s DGM label with the subject’s FIRST thalamus mask, because MindGlide does not output a thalamus-only label and its DGM label frequently contained caudate–thalamus bridges that prevented robust separation using automated morphological operations. FIRST was selected because, in visual inspection, its thalamus mask provided a spatial envelope that typically extended beyond the thalamic portion of MindGlide’s DGM label while excluding caudate-connected voxels, thereby minimizing the risk of clipping MindGlide-labeled thalamus. Nevertheless, because the final mask was constrained by FIRST’s mask, we cannot exclude some dependence of MindGlide-derived thalamus segmentations on FIRST boundary placement in individual cases. Second, our multi-sequence ensemble masks were generated via majority-voted or union across single-modality segmentations. A more consistent approach between two-sequence and three-sequence ensembles might further improve performance and could be explored in future work. Moreover, given its open-source status, it is possible to modify MindGlide’s CNN architecture with additional layers to accept multiple MRI inputs simultaneously, then fine-tune it on majority-voted ensemble labels.

Some caveats pertain to our clinical association analyses. We excluded FreeSurfer from the analyses based on the GT benchmarking results. FreeSurfer is widely used and was the only method evaluated here that offers a dedicated longitudinal pipeline. However, when compared to GT, FreeSurfer exhibited the largest mean bias and proportional bias relative to manual labels, without clear advantages in CCC/ICC or voxel-wise overlap compared with the methods retained for clinical analyses. To reduce analytical complexity and maintain interpretability, especially for longitudinal analyses with smaller sample sizes, we therefore restricted the clinical association analyses to a subset of methods chosen a priori based on GT agreement rather than on clinical-association results. This choice narrows the scope of the clinical comparison and does not address whether dedicated longitudinal pipelines provide added value for detecting clinically meaningful thalamic change; future work in larger longitudinal cohorts should explicitly evaluate longitudinal-specific approaches (including FreeSurfer’s longitudinal stream) against GT-consistent reference labels. Furthermore, in our longitudinal data, the limited magnitude of clinical change over the one-year follow-up interval constrains the interpretation of longitudinal associations. Small average changes in clinical scores, such as EDSS, reduce sensitivity to detect robust structure–function relationships and necessitate cautious interpretation of effect sizes.

Additionally, some limitations concern data availability. Manual GT labels were available only for 50 patients, and only for the baseline timepoint, which limited generalizability and precluded the evaluation of longitudinal thalamus atrophy against GT segmentations. GT labels were manually segmented using T1w and FLAIR images; though this is standard procedure in the field, using R1 maps could potentially improve manual segmentations of the thalamus. Consequently, when comparing automated R1 segmentations to GT masks segmented using T1w and FLAIR images, deviations from GT may not necessarily indicate inferior performance, but could instead reflect modality-specific differences in anatomical contrasts. Moreover, we did not perform external validation on multi-scanner or multi-center data; all scans were acquired on the same scanner platform, so the generalizability of these findings to other MRI systems has not been tested.

Finally, while the quantitative stability and reduced scanner-dependence of MPM make it particularly advantageous for longitudinal comparisons, the effects observed in this study may have been limited by the spatial resolution of our MPM data (1.6 mm isotropic resolution, compared to 0.8 mm for T1w and FLAIR). This resolution was chosen to reduce scanner time as well as improve signal-to-noise ratio, thus increasing the reliability of quantitative parameter estimations. Nevertheless, it is possible that a higher spatial fidelity may have further improved the R1-inclusive thalamus masks and their clinical relevance. It is also worth noting that three rigid co-registration schemes were evaluated to ensure robust anatomical alignment of R1 to T1w images. Although our QC-based procedure was applied consistently across participants, alternative alignment strategies may yield subtle differences and warrant evaluation in future multimodal segmentation studies. Moreover, this study focused on R1 maps as a proof-of-concept quantitative sequence. From a practical standpoint, MT ratio or MT saturation maps as part of the MPM framework might provide similar contrast benefits to R1. Clinically, while R1 can track both myelin and iron, it is less sensitive than MTsat for myelin, and less sensitive than R2* for iron (Weiskopf et al., 2013, Tabelow et al., 2019, Lorio et al., 2016); both sequences could therefore offer additional information beyond R1 that could improve DGM segmentations in relevant patient cohorts. Lastly, although we did not include diffusion-weighted imaging (DWI) in the current study, some evidence suggests that the use of fractional anisotropy maps may establish more robust lateral boundaries in thalamus segmentations (Bisecco et al., 2015, Storelli et al., 2023). Future work could explore a more extensive multimodal approach where structural (T1, FLAIR), microstructural (different MPM-derived maps), and tract-related (DWI) information is used to constrain thalamic boundary segmentations more accurately.

## 6 Conclusions

Our results highlight the advantage of nnU-net-based segmentation methods over atlas-constrained approaches in the context of small DGM structures like the thalamus. In particular, we found that DBSegment achieved the highest spatial agreement with GT, whereas MindGlide produced volumes with the best agreement to GT volumes and showed the strongest correlations with clinical outcome measures. In addition, though differences were small, we provide preliminary evidence that adopting an ensemble approach to thalamus segmentation incorporating T1w, FLAIR, and quantitative R1 images may improve the longitudinal clinical relevance of thalamus volumetric measurements. Future work will build on these findings to further improve the automatic segmentation of the thalamus and other DGM structures in the context of demyelination and neurodegeneration in patients with neuroinflammatory diseases like MS.

## CRediT authorship contribution statement

**Omar Angelo Ibrahim:** Writing – review & editing, Writing – original draft, Visualization, Validation, Software, Methodology, Investigation, Formal analysis, Data curation. **Henri Trang:** Software, Methodology. **Qianlan Chen:** Methodology. **Lara Zimmermann:** Data curation. **Alexander U. Brandt:** Funding acquisition. **Tatiana Usnich:** Data curation. **Stefano Magon:** Writing – review & editing, Project administration. **Muhamed Barakovic:** Writing – review & editing, Project administration. **Jens Wuerfel:** Writing – review & editing, Project administration, Conceptualization. **Friedemann Paul:** Writing – review & editing, Project administration, Funding acquisition, Conceptualization. **Martin Bauer:** Writing – review & editing, Supervision, Software, Project administration, Methodology, Investigation, Data curation, Conceptualization. **Lina Anderhalten:** Writing – review & editing, Writing – original draft, Supervision, Project administration, Methodology, Investigation, Data curation, Conceptualization.

## Funding

This research project was partly funded by F. Hoffmann-La Roche Ltd., Basel, Switzerland, as part of the Integrative Neuroscience Collaborations Network. Apart from the scholarly work performed by sponsor-affiliated co-authors (see CRediT authorship contribution statement), the funder had no additional role in the study design, data collection, data analysis, data interpretation, manuscript preparation, or the decision to submit.

## Data Availability

The data that has been used is confidential.
