# Supplementary material for: Microstructure-informed deep learning improves thalamic atrophy segmentation and clinical associations in multiple sclerosis and related neuroimmunological diseases
Source: Neuroimage Clin. 2026 Mar 2;49:103982. doi: 10.1016/j.nicl.2026.103982 (PMC12993160; doi:10.1016/j.nicl.2026.103982)
Supplement: Supplementary Data 1 [file mmc1.docx]

**Microstructure-Informed Deep Learning Improves Thalamic Atrophy Segmentation and Clinical Associations in Multiple Sclerosis and Related Neuroimmunological Diseases**

**Supplementary Material**

**SM.A – Distribution of Baseline Characteristics in the Study Populations**


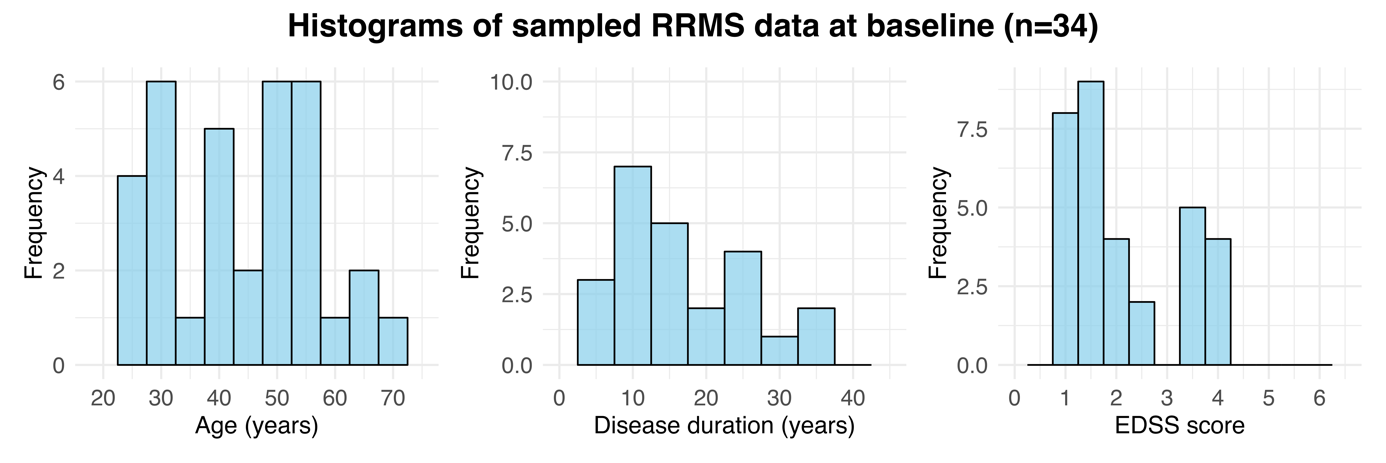


**A)**

**
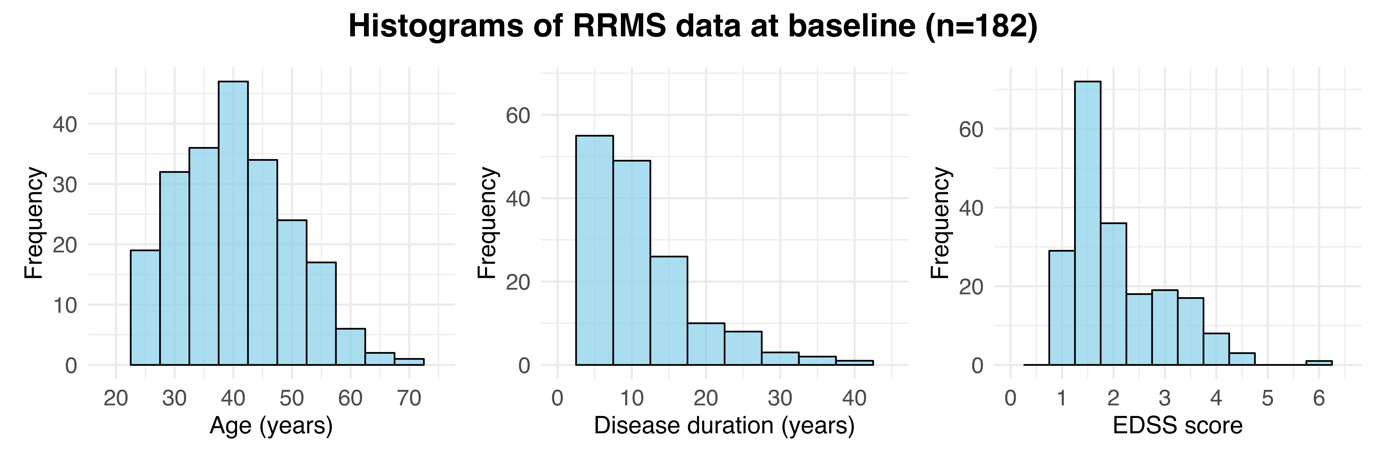
**

**B)**

**Figure S1.** Baseline distributions of age, disease duration and EDSS score of RRMS patients in **a)** the sampled GT population (n=34) and **b)** the overall cross-sectional FAS population (n=203).


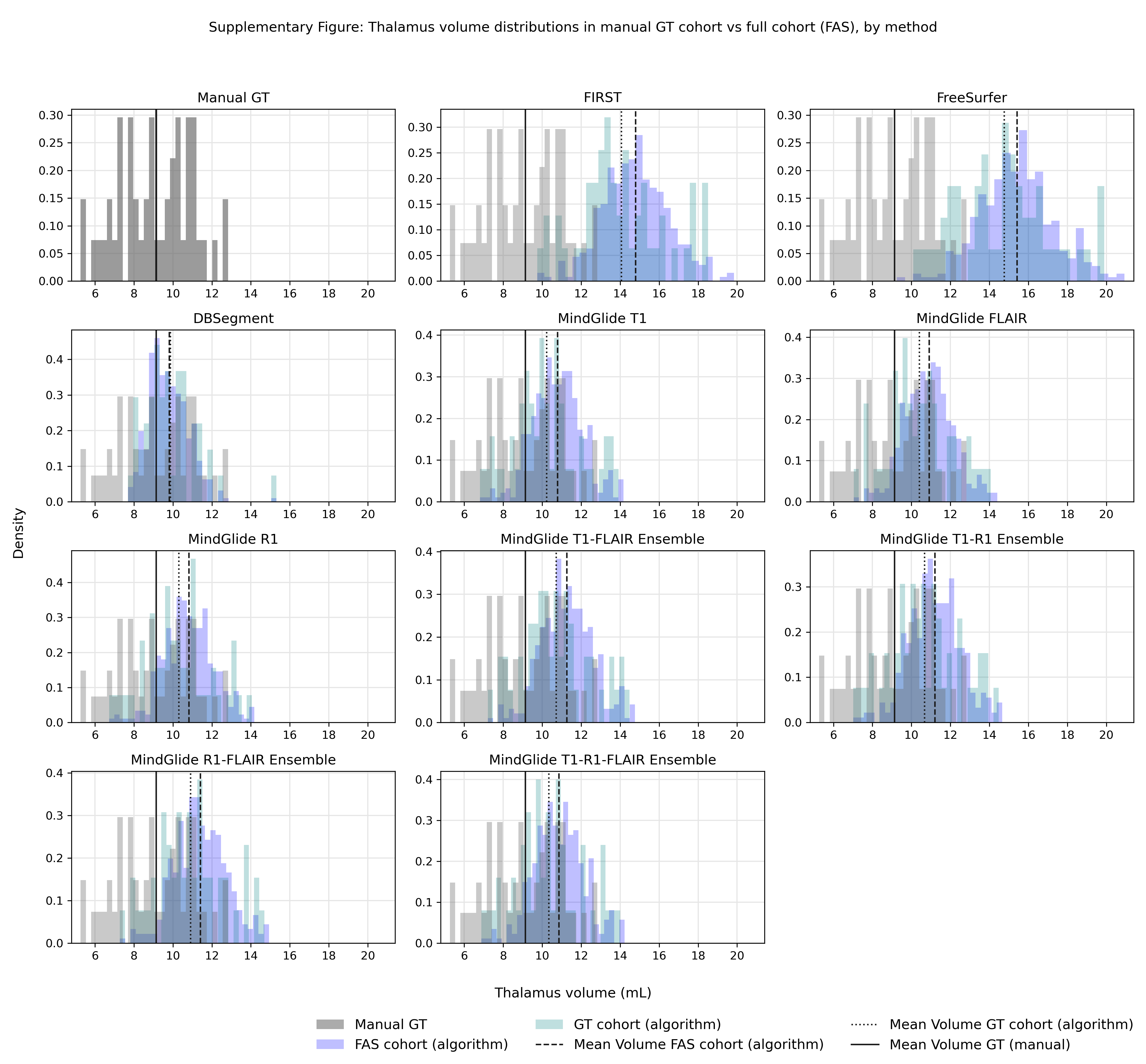
**Figure S2.** Baseline distributions and mean thalamus volumes for GT cohort (n=50) and full cross-sectional FAS cohort (n=321).

**Table S1. Demographic and baseline clinical characteristics of patients with GT labels**

|  | **Total** | **RRMS** | **PMS** |
| --- | --- | --- | --- |
| **Sample size (n)** | 50 | 34 | 16 |
| **Age (years)** | 48.4 ± 13.0 | 44.2 ± 12.3 | 57.3 ± 9.5 |
| **Age range (years)** | 26.0–72.7 | 26.0–68.8 | 36.2–72.7 |
| **Female / Male (n)** | 25 / 25 | 18 / 16 | 7 / 9 |
| **IMT (n)** | 23 | 16 | 7 |
| **EDSS** | 3.00 [1.50–4.00] | 1.50 [1.00–3.38] | 5.50 [3.50–6.00] |
| **T25FW (s)** | 4.40 [3.88–6.00] | 4.12 [3.75–4.69] | 6.60 [5.05–7.65] |
| **9HPT (s)** | 21.19 [18.52–26.40] | 19.83 [18.10–23.78] | 24.46 [21.89–30.11] |
| **SDMT** | 53.39 ± 13.05 | 57.00 ± 11.80 | 45.94 ± 12.66 |
| **Disease duration (years)** | 16.70 ± 12.95 | 13.52 ± 11.65 | 23.92 ± 13.21 |

**Note:** Baseline demographics and clinical outcome variables are reported as median [IQR] if skewed in distribution (T25FW and 9HPT) or ordinal (EDSS), otherwise as mean ± SD. *IMT = On Immunotherapy.*

**Table S2. Demographic and baseline clinical characteristics of patients in the FAS population for cross-sectional outcome analysis**

|  | **Total** | **Healthy** | **RIS/CIS** | **RRMS** | **PMS** | **NMOSD** | **MOGAD** |
| --- | --- | --- | --- | --- | --- | --- | --- |
| **Sample size (n)** | 321 | 10 | 20 | 203 | 16 | 42 | 30 |
| **Age (years)** | 43.4 ± 12.4 | 46.6 ± 14.5 | 38.0 ± 10.3 | 40.9 ± 9.8 | 57.3 ± 9.5 | 53.8 ± 15.2 | 41.5 ± 13.4 |
| **Age range (years)** | 18.6–79.3 | 25.2–69.7 | 19.9–61.7 | 18.6–68.8 | 36.2–72.7 | 20.2–79.3 | 20.7–71.3 |
| **Female / Male (n)** | 217 / 104 | 6 / 4 | 13 / 7 | 139 / 64 | 7 / 9 | 35 / 7 | 17 / 13 |
| **IMT (n)** | 169 | 0 | 5 | 107 | 7 | 30 | 19 |
| **EDSS** | 2.00 [1.50–3.00] | 1.50 [0.75–2.25] | 1.50 [0.00–1.50] | 1.50 [1.50–2.50] | 5.50 [3.50–6.00] | 3.00 [2.00–4.00] | 2.00 [1.50–3.50] |
| **T25FW (s)** | 4.20 [3.80–4.75] | 3.85 [3.76–4.31] | 3.80 [3.62–4.25] | 4.15 [3.80–4.50] | 6.60 [5.05–7.65] | 4.88 [4.45–7.02] | 4.35 [3.84–4.82] |
| **9HPT (s)** | 19.82 [17.89–22.06] | 18.70 [16.23–19.44] | 19.02 [17.80–20.41] | 19.30 [17.70–21.50] | 24.46 [21.89–30.11] | 21.25 [19.00–23.36] | 20.06 [18.78–23.09] |
| **SDMT** | 57.55 ± 13.03 | 62.44 ± 6.64 | 56.95 ± 14.02 | 59.62 ± 11.76 | 45.94 ± 12.66 | 53.38 ± 14.56 | 53.86 ± 15.55 |
| **Disease duration (years)** | 9.19 ± 8.90 | 0 | 3.69 ± 4.25 | 8.81 ± 7.97 | 23.92 ± 13.21 | 11.36 ± 8.15 | 5.11 ± 7.44 |

**Note:** Baseline demographics and clinical outcome variables are reported as median [IQR] if skewed in distribution (T25FW and 9HPT) or ordinal (EDSS), otherwise as mean ± SD. *IMT = On Immunotherapy.*

**Table S3. Demographic and baseline clinical characteristics of patients in the FAS population for longitudinal outcome analysis**

|  | **Total** | **Healthy** | **RIS/CIS** | **RRMS** | **PMS** | **NMOSD** | **MOGAD** |
| --- | --- | --- | --- | --- | --- | --- | --- |
| **Sample size (n)** | 234 | 7 | 14 | 162 | 14 | 24 | 13 |
| **Age (years)** | 43.7 ± 12.0 | 44.5 ± 14.2 | 38.9 ± 10.6 | 41.5 ± 9.5 | 56.8 ± 10.1 | 56.5 ± 14.6 | 39.3 ± 12.9 |
| **Age range (years)** | 18.6–79.3 | 25.2–69.7 | 21.9–61.7 | 18.6–68.8 | 36.2–72.7 | 20.2–79.3 | 20.7–65.0 |
| **Female / Male (n)** | 158 / 76 | 5 / 2 | 10 / 4 | 112 / 50 | 6 / 8 | 19 / 5 | 6 / 7 |
| **IMT (n)** | 121 | 0 | 3 | 85 | 7 | 16 | 10 |
| **EDSS** | 2.00 [1.50–3.00] | 1.50 [0.75–2.25] | 1.25 [0.25–1.50] | 1.50 [1.50–2.50] | 5.50 [3.50–6.00] | 3.50 [2.50–4.12] | 2.00 [1.50–3.50] |
| **T25FW (s)** | 4.15 [3.85–4.70] | 3.85 [3.77–3.95] | 3.92 [3.66–4.17] | 4.15 [3.85–4.49] | 5.85 [4.90–7.47] | 4.88 [4.50–7.29] | 4.30 [3.75–4.85] |
| **9HPT (s)** | 19.85 [17.90–22.30] | 16.40 [16.12–20.17] | 19.55 [17.96–20.52] | 19.32 [17.73–21.43] | 23.46 [21.53–29.56] | 21.42 [19.89–25.35] | 20.48 [18.70–21.23] |
| **SDMT** | 57.77 ± 12.99 | 62.43 ± 7.52 | 58.57 ± 16.36 | 59.48 ± 11.84 | 48.50 ± 10.63 | 50.87 ± 13.95 | 55.25 ± 18.55 |
| **Disease**  **duration (years)** | 9.7 ± 8.9 | 0 | 3.8 ± 4.5 | 9.3 ± 7.9 | 22.9 ± 13.7 | 12.4 ± 8.5 | 4.1 ± 5.2 |

**Note:** Baseline demographics and clinical outcome variables are reported as median [IQR] if skewed in distribution (T25FW and 9HPT) or ordinal (EDSS), otherwise as mean ± SD. *IMT = On Immunotherapy.*

**SM.B - Voxel-wise Agreement with GT**

**DSC, Precision and Sensitivity Calculations**
For each subject, algorithm mask A and ground-truth mask G were binarized and compared voxel-wise. The below equations were then used to compute True Positives (TP), False Positives (FP) and False Negatives (FN) as voxel counts:

TP = ∣A∩G∣

FP = ∣A∖G∣

FN = ∣G∖A∣

Subsequently, Dice Similarity Coefficients (DSC), Precision, and Sensitivity were computed using the following equations:

DSC = 2TP / (2TP + FP + FN)

Precision = TP / (TP + FP)

Sensitivity = TP / (TP + FN)

**Table S4.** *Pairwise Comparison Tables of DSC, Precision and Sensitivity*

**a. Dice Similarity Coefficients (DSC)**

| **Method 1** | **Method 2** | **n** | **z** | **p** | **p_corr** |
| --- | --- | --- | --- | --- | --- |
| FreeSurfer | DBSegment | 50 | -5.49 | 0.000 | 0.000 |
| FreeSurfer | FIRST | 50 | -4.78 | 0.000 | 0.000 |
| FreeSurfer | Mindglide R1 | 49 | -3.65 | 0.000 | 0.000 |
| FreeSurfer | Mindglide T1-R1 Ensemble | 49 | -3.26 | 0.001 | 0.001 |
| FreeSurfer | Mindglide R1-FLAIR Ensemble | 49 | -2.78 | 0.005 | 0.007 |
| FreeSurfer | Mindglide T1-R1-FLAIR Ensemble | 49 | -2.17 | 0.029 | 0.040 |
| FreeSurfer | Mindglide T1 | 50 | -2.01 | 0.044 | 0.055 |
| FreeSurfer | Mindglide T1-FLAIR Ensemble | 50 | -1.52 | 0.130 | 0.154 |
| FreeSurfer | Mindglide FLAIR | 50 | -1.02 | 0.314 | 0.362 |
| FIRST | DBSegment | 50 | -4.50 | 0.000 | 0.000 |
| FIRST | Mindglide FLAIR | 50 | -2.02 | 0.043 | 0.055 |
| FIRST | Mindglide T1-FLAIR Ensemble | 50 | -1.68 | 0.093 | 0.113 |
| FIRST | Mindglide T1 | 50 | -0.94 | 0.352 | 0.396 |
| FIRST | Mindglide T1-R1-FLAIR Ensemble | 49 | -0.81 | 0.424 | 0.465 |
| FIRST | Mindglide T1-R1 Ensemble | 49 | -0.27 | 0.790 | 0.805 |
| FIRST | Mindglide R1 | 49 | -0.79 | 0.435 | 0.466 |
| FIRST | Mindglide R1-FLAIR Ensemble | 49 | -0.36 | 0.723 | 0.756 |
| DBSegment | Mindglide FLAIR | 50 | -5.57 | 0.000 | 0.000 |
| DBSegment | Mindglide T1-FLAIR Ensemble | 50 | -5.50 | 0.000 | 0.000 |
| DBSegment | Mindglide T1 | 50 | -5.45 | 0.000 | 0.000 |
| DBSegment | Mindglide T1-R1-FLAIR Ensemble | 49 | -5.33 | 0.000 | 0.000 |
| DBSegment | Mindglide R1-FLAIR Ensemble | 49 | -5.12 | 0.000 | 0.000 |
| DBSegment | Mindglide T1-R1 Ensemble | 49 | -5.01 | 0.000 | 0.000 |
| DBSegment | Mindglide R1 | 49 | -4.82 | 0.000 | 0.000 |
| Mindglide T1 | Mindglide R1 | 49 | -5.80 | 0.000 | 0.000 |
| Mindglide T1 | Mindglide T1-R1 Ensemble | 49 | -5.62 | 0.000 | 0.000 |
| Mindglide T1 | Mindglide FLAIR | 50 | -4.87 | 0.000 | 0.000 |
| Mindglide T1 | Mindglide T1-FLAIR Ensemble | 50 | -4.05 | 0.000 | 0.000 |
| Mindglide T1 | Mindglide R1-FLAIR Ensemble | 49 | -2.14 | 0.032 | 0.042 |
| Mindglide T1 | Mindglide T1-R1-FLAIR Ensemble | 49 | -0.25 | 0.805 | 0.805 |
| Mindglide FLAIR | Mindglide R1-FLAIR Ensemble | 49 | -6.05 | 0.000 | 0.000 |
| Mindglide FLAIR | Mindglide T1-R1 Ensemble | 49 | -6.02 | 0.000 | 0.000 |
| Mindglide FLAIR | Mindglide R1 | 49 | -5.94 | 0.000 | 0.000 |
| Mindglide FLAIR | Mindglide T1-R1-FLAIR Ensemble | 49 | -5.48 | 0.000 | 0.000 |
| Mindglide FLAIR | Mindglide T1-FLAIR Ensemble | 50 | -4.39 | 0.000 | 0.000 |
| Mindglide R1 | Mindglide T1-FLAIR Ensemble | 49 | -5.95 | 0.000 | 0.000 |
| Mindglide R1 | Mindglide T1-R1-FLAIR Ensemble | 49 | -5.90 | 0.000 | 0.000 |
| Mindglide R1 | Mindglide R1-FLAIR Ensemble | 49 | -5.33 | 0.000 | 0.000 |
| Mindglide R1 | Mindglide T1-R1 Ensemble | 49 | -4.61 | 0.000 | 0.000 |
| Mindglide T1-FLAIR Ensemble | Mindglide T1-R1 Ensemble | 49 | -6.06 | 0.000 | 0.000 |
| Mindglide T1-FLAIR Ensemble | Mindglide R1-FLAIR Ensemble | 49 | -6.06 | 0.000 | 0.000 |
| Mindglide T1-FLAIR Ensemble | Mindglide T1-R1-FLAIR Ensemble | 49 | -4.93 | 0.000 | 0.000 |
| Mindglide T1-R1 Ensemble | Mindglide T1-R1-FLAIR Ensemble | 49 | -6.08 | 0.000 | 0.000 |
| Mindglide T1-R1 Ensemble | Mindglide R1-FLAIR Ensemble | 49 | -5.07 | 0.000 | 0.000 |
| Mindglide R1-FLAIR Ensemble | Mindglide T1-R1-FLAIR Ensemble | 49 | -2.86 | 0.004 | 0.005 |

**b. Precision**

| **Method 1** | **Method 2** | **n** | **z** | **p** | **p_corr** |
| --- | --- | --- | --- | --- | --- |
| FreeSurfer | Mindglide T1 | 50 | -6.15 | 0.000000 | 0.000000 |
| FreeSurfer | Mindglide FLAIR | 50 | -6.15 | 0.000000 | 0.000000 |
| FreeSurfer | Mindglide T1-FLAIR Ensemble | 50 | -6.15 | 0.000000 | 0.000000 |
| FreeSurfer | Mindglide R1 | 49 | -6.09 | 0.000000 | 0.000000 |
| FreeSurfer | Mindglide T1-R1 Ensemble | 49 | -6.09 | 0.000000 | 0.000000 |
| FreeSurfer | Mindglide R1-FLAIR Ensemble | 49 | -6.09 | 0.000000 | 0.000000 |
| FreeSurfer | Mindglide T1-R1-FLAIR Ensemble | 49 | -6.09 | 0.000000 | 0.000000 |
| FreeSurfer | DBSegment | 50 | -6.07 | 0.000000 | 0.000000 |
| FreeSurfer | FIRST | 50 | -4.69 | 0.000000 | 0.000000 |
| FIRST | Mindglide T1 | 50 | -6.14 | 0.000000 | 0.000000 |
| FIRST | Mindglide FLAIR | 50 | -6.13 | 0.000000 | 0.000000 |
| FIRST | Mindglide R1 | 49 | -6.08 | 0.000000 | 0.000000 |
| FIRST | Mindglide T1-R1-FLAIR Ensemble | 49 | -6.07 | 0.000000 | 0.000000 |
| FIRST | Mindglide T1-R1 Ensemble | 49 | -6.07 | 0.000000 | 0.000000 |
| FIRST | Mindglide T1-FLAIR Ensemble | 50 | -6.11 | 0.000000 | 0.000000 |
| FIRST | Mindglide R1-FLAIR Ensemble | 49 | -6.04 | 0.000000 | 0.000000 |
| FIRST | DBSegment | 50 | -5.88 | 0.000000 | 0.000000 |
| DBSegment | Mindglide T1-FLAIR Ensemble | 50 | -4.85 | 0.000000 | 0.000000 |
| DBSegment | Mindglide R1-FLAIR Ensemble | 49 | -4.77 | 0.000000 | 0.000000 |
| DBSegment | Mindglide FLAIR | 50 | -4.73 | 0.000000 | 0.000000 |
| DBSegment | Mindglide T1-R1 Ensemble | 49 | -4.40 | 0.000000 | 0.000000 |
| DBSegment | Mindglide T1 | 50 | -4.23 | 0.000010 | 0.000010 |
| DBSegment | Mindglide T1-R1-FLAIR Ensemble | 49 | -4.24 | 0.000010 | 0.000010 |
| DBSegment | Mindglide R1 | 49 | -3.94 | 0.000040 | 0.000041 |
| Mindglide T1 | Mindglide T1-FLAIR Ensemble | 50 | -6.15 | 0.000000 | 0.000000 |
| Mindglide T1 | Mindglide R1-FLAIR Ensemble | 49 | -6.09 | 0.000000 | 0.000000 |
| Mindglide T1 | Mindglide FLAIR | 50 | -6.13 | 0.000000 | 0.000000 |
| Mindglide T1 | Mindglide T1-R1 Ensemble | 49 | -6.04 | 0.000000 | 0.000000 |
| Mindglide T1 | Mindglide T1-R1-FLAIR Ensemble | 49 | -5.66 | 0.000000 | 0.000000 |
| Mindglide T1 | Mindglide R1 | 49 | -5.43 | 0.000000 | 0.000000 |
| Mindglide FLAIR | Mindglide T1-FLAIR Ensemble | 50 | -6.15 | 0.000000 | 0.000000 |
| Mindglide FLAIR | Mindglide R1 | 49 | -6.08 | 0.000000 | 0.000000 |
| Mindglide FLAIR | Mindglide T1-R1-FLAIR Ensemble | 49 | -6.05 | 0.000000 | 0.000000 |
| Mindglide FLAIR | Mindglide R1-FLAIR Ensemble | 49 | -5.77 | 0.000000 | 0.000000 |
| Mindglide FLAIR | Mindglide T1-R1 Ensemble | 49 | -5.07 | 0.000000 | 0.000000 |
| Mindglide R1 | Mindglide R1-FLAIR Ensemble | 49 | -6.09 | 0.000000 | 0.000000 |
| Mindglide R1 | Mindglide T1-R1-FLAIR Ensemble | 49 | -6.09 | 0.000000 | 0.000000 |
| Mindglide R1 | Mindglide T1-FLAIR Ensemble | 49 | -6.09 | 0.000000 | 0.000000 |
| Mindglide R1 | Mindglide T1-R1 Ensemble | 49 | -6.09 | 0.000000 | 0.000000 |
| Mindglide T1-FLAIR Ensemble | Mindglide T1-R1-FLAIR Ensemble | 49 | -6.09 | 0.000000 | 0.000000 |
| Mindglide T1-FLAIR Ensemble | Mindglide T1-R1 Ensemble | 49 | -6.08 | 0.000000 | 0.000000 |
| Mindglide T1-FLAIR Ensemble | Mindglide R1-FLAIR Ensemble | 49 | -3.65 | 0.000160 | 0.000160 |
| Mindglide T1-R1 Ensemble | Mindglide R1-FLAIR Ensemble | 49 | -6.09 | 0.000000 | 0.000000 |
| Mindglide T1-R1 Ensemble | Mindglide T1-R1-FLAIR Ensemble | 49 | -5.84 | 0.000000 | 0.000000 |
| Mindglide R1-FLAIR Ensemble | Mindglide T1-R1-FLAIR Ensemble | 49 | -6.09 | 0.000000 | 0.000000 |

**c. Sensitivity**

| **Method 1** | **Method 2** | **n** | **z** | **p** | **p_corr** |
| --- | --- | --- | --- | --- | --- |
| FreeSurfer | Mindglide FLAIR | 50 | -6.15 | 0.00000 | 0.00000 |
| FreeSurfer | Mindglide T1 | 50 | -6.15 | 0.00000 | 0.00000 |
| FreeSurfer | DBSegment | 50 | -6.15 | 0.00000 | 0.00000 |
| FreeSurfer | Mindglide T1-FLAIR Ensemble | 50 | -6.15 | 0.00000 | 0.00000 |
| FreeSurfer | Mindglide T1-R1-FLAIR Ensemble | 49 | -6.09 | 0.00000 | 0.00000 |
| FreeSurfer | Mindglide R1-FLAIR Ensemble | 49 | -6.09 | 0.00000 | 0.00000 |
| FreeSurfer | Mindglide T1-R1 Ensemble | 49 | -6.09 | 0.00000 | 0.00000 |
| FreeSurfer | Mindglide R1 | 49 | -6.09 | 0.00000 | 0.00000 |
| FreeSurfer | FIRST | 50 | -1.61 | 0.10953 | 0.11462 |
| FIRST | Mindglide T1-FLAIR Ensemble | 50 | -6.15 | 0.00000 | 0.00000 |
| FIRST | Mindglide FLAIR | 50 | -6.15 | 0.00000 | 0.00000 |
| FIRST | Mindglide T1 | 50 | -6.15 | 0.00000 | 0.00000 |
| FIRST | DBSegment | 50 | -6.15 | 0.00000 | 0.00000 |
| FIRST | Mindglide T1-R1-FLAIR Ensemble | 49 | -6.09 | 0.00000 | 0.00000 |
| FIRST | Mindglide T1-R1 Ensemble | 49 | -6.09 | 0.00000 | 0.00000 |
| FIRST | Mindglide R1 | 49 | -6.09 | 0.00000 | 0.00000 |
| FIRST | Mindglide R1-FLAIR Ensemble | 49 | -6.09 | 0.00000 | 0.00000 |
| DBSegment | Mindglide FLAIR | 50 | -5.85 | 0.00000 | 0.00000 |
| DBSegment | Mindglide T1 | 50 | -5.84 | 0.00000 | 0.00000 |
| DBSegment | Mindglide T1-R1-FLAIR Ensemble | 49 | -5.67 | 0.00000 | 0.00000 |
| DBSegment | Mindglide R1 | 49 | -5.31 | 0.00000 | 0.00000 |
| DBSegment | Mindglide T1-FLAIR Ensemble | 50 | -5.39 | 0.00000 | 0.00000 |
| DBSegment | Mindglide T1-R1 Ensemble | 49 | -4.80 | 0.00000 | 0.00000 |
| DBSegment | Mindglide R1-FLAIR Ensemble | 49 | -4.43 | 0.00000 | 0.00000 |
| Mindglide T1 | Mindglide T1-FLAIR Ensemble | 50 | -6.15 | 0.00000 | 0.00000 |
| Mindglide T1 | Mindglide R1-FLAIR Ensemble | 49 | -6.09 | 0.00000 | 0.00000 |
| Mindglide T1 | Mindglide T1-R1 Ensemble | 49 | -6.09 | 0.00000 | 0.00000 |
| Mindglide T1 | Mindglide R1 | 49 | -5.49 | 0.00000 | 0.00000 |
| Mindglide T1 | Mindglide T1-R1-FLAIR Ensemble | 49 | -4.35 | 0.00000 | 0.00000 |
| Mindglide T1 | Mindglide FLAIR | 50 | -0.03 | 0.97710 | 0.97710 |
| Mindglide FLAIR | Mindglide T1-FLAIR Ensemble | 50 | -6.15 | 0.00000 | 0.00000 |
| Mindglide FLAIR | Mindglide R1-FLAIR Ensemble | 49 | -6.09 | 0.00000 | 0.00000 |
| Mindglide FLAIR | Mindglide T1-R1 Ensemble | 49 | -5.93 | 0.00000 | 0.00000 |
| Mindglide FLAIR | Mindglide R1 | 49 | -4.61 | 0.00000 | 0.00000 |
| Mindglide FLAIR | Mindglide T1-R1-FLAIR Ensemble | 49 | -2.74 | 0.00550 | 0.00589 |
| Mindglide R1 | Mindglide T1-R1 Ensemble | 49 | -6.09 | 0.00000 | 0.00000 |
| Mindglide R1 | Mindglide R1-FLAIR Ensemble | 49 | -6.09 | 0.00000 | 0.00000 |
| Mindglide R1 | Mindglide T1-R1-FLAIR Ensemble | 49 | -5.29 | 0.00000 | 0.00000 |
| Mindglide R1 | Mindglide T1-FLAIR Ensemble | 49 | -0.37 | 0.89393 | 0.91425 |
| Mindglide T1-FLAIR Ensemble | Mindglide T1-R1-FLAIR Ensemble | 49 | -6.09 | 0.00000 | 0.00000 |
| Mindglide T1-FLAIR Ensemble | Mindglide R1-FLAIR Ensemble | 49 | -6.09 | 0.00000 | 0.00000 |
| Mindglide T1-FLAIR Ensemble | Mindglide T1-R1 Ensemble | 49 | -4.74 | 0.00000 | 0.00000 |
| Mindglide T1-R1 Ensemble | Mindglide T1-R1-FLAIR Ensemble | 49 | -6.09 | 0.00000 | 0.00000 |
| Mindglide T1-R1 Ensemble | Mindglide R1-FLAIR Ensemble | 49 | -5.00 | 0.00000 | 0.00000 |
| Mindglide R1-FLAIR Ensemble | Mindglide T1-R1-FLAIR Ensemble | 49 | -6.09 | 0.00000 | 0.00000 |

***Note:*** All reported p_corr values are Benjamini-Hochberg-adjusted (a-c).

**SM.C - Volumetric Agreement with GT**

**Table S5. Algorithm vs GT Volume Regression Statistics**

| **Method** | **β (Alg~GT)** | **95% CI** | **Intercept (mL)** | **SD residuals (mL)** |
| --- | --- | --- | --- | --- |
| **FreeSurfer** | 0.930 | [0.62, 1.24] | 6.28 | 1.68 |
| **FIRST** | 0.783 | [0.47, 1.10] | 6.93 | 1.67 |
| **DBSegment** | 0.370 | [0.22, 0.52] | 6.50 | 1.17 |
| **MindGlide T1** | 0.673 | [0.44, 0.91] | 4.10 | 1.21 |
| **MindGlide FLAIR** | 0.629 | [0.40, 0.85] | 4.67 | 1.19 |
| **MindGlide R1** | 0.659 | [0.43, 0.88] | 4.29 | 1.16 |
| **MindGlide T1-FLAIR Ensemble** | 0.668 | [0.43, 0.91] | 4.63 | 1.24 |
| **MindGlide T1-R1 Ensemble** | 0.688 | [0.45, 0.92] | 4.37 | 1.22 |
| **MindGlide R1-FLAIR Ensemble** | 0.677 | [0.44, 0.91] | 4.72 | 1.24 |
| **MindGlide T1-R1-FLAIR Ensemble** | 0.660 | [0.43, 0.89] | 4.31 | 1.20 |

**Table S6. Bland-Altman and Bias Statistics**

| **Method** | **Mean Bias (mL)** | **SD (mL)** | **95% LoA** | **β (error~mean)** | **95% CI** |
| --- | --- | --- | --- | --- | --- |
| **FreeSurfer** | 5.65 | 1.69 | [2.34, 8.95] | 0.28 | [0.05, 0.51] |
| **FIRST** | 4.94 | 1.72 | [1.57, 8.31] | 0.19 | [-0.07, 0.45] |
| **DBSegment** | 0.75 | 1.68 | [-2.54, 4.05] | -0.43 | [-0.75, -0.11] |
| **MindGlide T1** | 1.11 | 1.37 | [-1.57, 3.79] | -0.09 | [-0.32, 0.14] |
| **MindGlide FLAIR** | 1.28 | 1.38 | [-1.43, 3.99] | -0.14 | [-0.38, 0.09] |
| **MindGlide R1** | 1.17 | 1.33 | [-1.44, 3.77] | -0.13 | [-0.36, 0.10] |
| **MindGlide T1-FLAIR Ensemble** | 1.59 | 1.40 | [-1.14, 4.33] | -0.08 | [-0.32, 0.16] |
| **MindGlide T1-R1 Ensemble** | 1.53 | 1.36 | [-1.14, 4.19] | -0.07 | [-0.30, 0.16] |
| **MindGlide R1-FLAIR Ensemble** | 1.77 | 1.38 | [-0.94, 4.48] | -0.08 | [-0.31, 0.16] |
| **MindGlide T1-R1-FLAIR Ensemble** | 1.20 | 1.36 | [-1.47, 3.87] | -0.11 | [-0.34, 0.12] |

**SM.D - Clinical Outcome Associations**

1. **Main Analysis**

**Table S7. Mass Univariate Analysis Results – Cross-sectional Outcome Analysis**

| **Clinical Outcome** | **Predictor (thalamus volume in mL)** | **n** | **β** | **SE** | **t** | **p** | **p_corr** | **R^2^** |
| --- | --- | --- | --- | --- | --- | --- | --- | --- |
| **EDSS** | FIRST | 309 | -0.191 | 0.061 | -3.132 | 0.0017 | 0.0024 | 0.320 |
|  | DBSegment | 309 | -0.137 | 0.111 | -1.237 | 0.2161 | 0.2210 | 0.294 |
|  | MindGlide T1 | 309 | -0.257 | 0.074 | -3.457 | 0.0005 | 0.0009 | 0.322 |
|  | MindGlide FLAIR | 309 | -0.275 | 0.078 | -3.524 | 0.0004 | 0.0008 | 0.325 |
|  | MindGlide R1 | 309 | -0.239 | 0.075 | -3.169 | 0.0015 | 0.0022 | 0.318 |
|  | MindGlide T1-FLAIR Ensemble | 309 | -0.253 | 0.073 | -3.458 | 0.0005 | 0.0009 | 0.323 |
|  | MindGlide T1-R1 Ensemble | 309 | -0.239 | 0.073 | -3.296 | 0.0010 | 0.0015 | 0.320 |
|  | MindGlide R1-FLAIR Ensemble | 309 | -0.244 | 0.073 | -3.357 | 0.0008 | 0.0012 | 0.321 |
|  | MindGlide T1-FLAIR-R1 Ensemble | 309 | -0.259 | 0.075 | -3.435 | 0.0006 | 0.0010 | 0.322 |
| **T25FW log(s)** | FIRST | 310 | -0.024 | 0.010 | -2.484 | 0.0135 | 0.0156 | 0.212 |
|  | DBSegment | 310 | -0.008 | 0.018 | -0.424 | 0.6720 | 0.6720 | 0.197 |
|  | MindGlide T1 | 310 | -0.032 | 0.012 | -2.535 | 0.0117 | 0.0143 | 0.213 |
|  | MindGlide FLAIR | 310 | -0.034 | 0.013 | -2.640 | 0.0087 | 0.0109 | 0.214 |
|  | MindGlide R1 | 310 | -0.028 | 0.013 | -2.253 | 0.0250 | 0.0261 | 0.209 |
|  | MindGlide T1-FLAIR Ensemble | 310 | -0.031 | 0.012 | -2.509 | 0.0126 | 0.0149 | 0.213 |
|  | MindGlide T1-R1 Ensemble | 310 | -0.028 | 0.012 | -2.339 | 0.0200 | 0.0214 | 0.210 |
|  | MindGlide R1-FLAIR Ensemble | 310 | -0.029 | 0.012 | -2.424 | 0.0159 | 0.0175 | 0.212 |
|  | MindGlide T1-FLAIR-R1 Ensemble | 310 | -0.031 | 0.013 | -2.467 | 0.0142 | 0.0159 | 0.212 |
| **9HPT log(s)** | FIRST | 315 | -0.040 | 0.007 | -5.890 | 0.0000 | 0.0000 | 0.286 |
|  | DBSegment | 315 | -0.043 | 0.014 | -3.132 | 0.0019 | 0.0026 | 0.231 |
|  | MindGlide T1 | 315 | -0.049 | 0.009 | -5.444 | 0.0000 | 0.0000 | 0.276 |
|  | MindGlide FLAIR | 315 | -0.051 | 0.009 | -5.583 | 0.0000 | 0.0000 | 0.279 |
|  | MindGlide R1 | 315 | -0.050 | 0.009 | -5.584 | 0.0000 | 0.0000 | 0.279 |
|  | MindGlide T1-FLAIR Ensemble | 315 | -0.047 | 0.009 | -5.434 | 0.0000 | 0.0000 | 0.276 |
|  | MindGlide T1-R1 Ensemble | 315 | -0.047 | 0.009 | -5.447 | 0.0000 | 0.0000 | 0.276 |
|  | MindGlide R1-FLAIR Ensemble | 315 | -0.047 | 0.009 | -5.482 | 0.0000 | 0.0000 | 0.277 |
|  | MindGlide T1-FLAIR-R1 Ensemble | 315 | -0.050 | 0.009 | -5.499 | 0.0000 | 0.0000 | 0.277 |
| **SDMT** | FIRST | 312 | 2.115 | 0.484 | 4.373 | 0.0000 | 0.0000 | 0.237 |
|  | DBSegment | 312 | 2.684 | 0.943 | 2.847 | 0.0047 | 0.0060 | 0.211 |
|  | MindGlide T1 | 312 | 2.627 | 0.628 | 4.184 | 0.0000 | 0.0001 | 0.233 |
|  | MindGlide FLAIR | 312 | 2.840 | 0.646 | 4.399 | 0.0000 | 0.0000 | 0.238 |
|  | MindGlide R1 | 312 | 2.647 | 0.628 | 4.213 | 0.0000 | 0.0001 | 0.234 |
|  | MindGlide T1-FLAIR Ensemble | 312 | 2.606 | 0.613 | 4.254 | 0.0000 | 0.0001 | 0.235 |
|  | MindGlide T1-R1 Ensemble | 312 | 2.486 | 0.608 | 4.086 | 0.0001 | 0.0001 | 0.232 |
|  | MindGlide R1-FLAIR Ensemble | 312 | 2.503 | 0.606 | 4.132 | 0.0000 | 0.0001 | 0.232 |
|  | MindGlide T1-FLAIR-R1 Ensemble | 312 | 2.667 | 0.634 | 4.208 | 0.0000 | 0.0001 | 0.234 |
| **Disease duration (years)** | FIRST | 310 | -1.519 | 0.381 | -3.991 | 0.0001 | 0.0001 | 0.277 |
|  | DBSegment | 310 | -1.910 | 0.675 | -2.830 | 0.0047 | 0.0060 | 0.246 |
|  | MindGlide T1 | 310 | -2.170 | 0.487 | -4.457 | 0.0000 | 0.0000 | 0.288 |
|  | MindGlide FLAIR | 310 | -2.185 | 0.502 | -4.349 | 0.0000 | 0.0000 | 0.285 |
|  | MindGlide R1 | 310 | -2.231 | 0.487 | -4.580 | 0.0000 | 0.0000 | 0.292 |
|  | MindGlide T1-FLAIR Ensemble | 310 | -2.089 | 0.476 | -4.392 | 0.0000 | 0.0000 | 0.286 |
|  | MindGlide T1-R1 Ensemble | 310 | -2.152 | 0.472 | -4.562 | 0.0000 | 0.0000 | 0.291 |
|  | MindGlide R1-FLAIR Ensemble | 310 | -2.120 | 0.469 | -4.521 | 0.0000 | 0.0000 | 0.290 |
|  | MindGlide T1-FLAIR-R1 Ensemble | 310 | -2.221 | 0.491 | -4.520 | 0.0000 | 0.0000 | 0.290 |

***Note:*** EDSS and disease duration OLS models were fitted with robust standard errors (HC3) due to heteroscedasticity. All remaining outcomes were fitted with standard OLS.

**Table S8. Delta Descriptive Statistics (clinical outcome change and thalamus volume loss between 1-year follow-up and baseline, longitudinal FAS (n=234))**

| **ΔThalamus Volume** | **Total (mean abs. (sd) / % (sd))** | **Healthy** | **RIS/CIS** | **RRMS** | **PMS** | **NMOSD** | **MOGAD** |
| --- | --- | --- | --- | --- | --- | --- | --- |
| **ΔThal – FIRST** | -92.03 (308.20) / -0.63 (2.11) | -104.35 (357.19) / -0.52 (2.21) | -76.00 (295.89) / -0.53 (1.79) | -78.01 (287.15) / -0.54 (1.99) | -154.48 (235.31) / -1.03 (1.64) | -144.47 (458.21) / -1.05 (3.15) | -108.18 (304.74) / -0.74 (2.03) |
| **ΔThal – DBSegment** | -6.54 (155.82) / -0.06 (1.55) | -64.05 (234.22) / -0.55 (2.14) | -6.84 (92.52) / -0.07 (0.93) | -0.84 (146.58) / -0.01 (1.49) | -30.79 (102.07) / -0.40 (0.93) | 1.07 (237.76) / 0.04 (2.31) | -15.91 (114.98) / -0.18 (1.15) |
| **ΔThal – Mindglide T1** | -34.79 (226.40) / -0.34 (2.11) | -54.69 (289.71) / -0.37 (2.39) | -21.74 (243.40) / -0.28 (1.91) | -32.39 (216.78) / -0.33 (2.06) | 2.70 (156.41) / 0.18 (1.65) | -52.85 (314.76) / -0.54 (2.95) | -66.46 (170.55) / -0.62 (1.51) |
| **ΔThal – Mindglide FLAIR** | -42.31 (216.29) / -0.41 (2.03) | 28.07 (231.51) / 0.36 (2.05) | -30.15 (276.39) / -0.35 (2.19) | -39.85 (206.02) / -0.40 (1.96) | -55.88 (170.54) / -0.45 (1.74) | -50.49 (278.37) / -0.55 (2.75) | -110.64 (193.00) / -0.98 (1.73) |
| **ΔThal – Mindglide R1** | -67.60 (222.45) / -0.65 (2.16) | -32.95 (199.67) / -0.19 (1.88) | -97.96 (244.27) / -0.91 (2.12) | -67.85 (225.66) / -0.67 (2.20) | -57.78 (225.69) / -0.51 (2.66) | -48.56 (208.74) / -0.53 (2.05) | -104.06 (229.56) / -0.94 (1.94) |
| **ΔThal – Mindglide T1+FLAIR Ensemble** | -51.82 (234.02) / -0.48 (2.11) | -13.73 (296.04) / 0.03 (2.43) | -84.61 (259.94) / -0.81 (1.99) | -44.15 (223.02) / -0.42 (2.03) | -37.85 (182.93) / -0.25 (1.88) | -85.98 (315.07) / -0.83 (2.89) | -92.98 (187.75) / -0.79 (1.60) |
| **ΔThal – Mindglide T1+R1 Ensemble** | -47.52 (219.58) / -0.45 (2.01) | -21.83 (279.02) / -0.04 (2.27) | -28.18 (269.59) / -0.34 (2.16) | -47.38 (218.19) / -0.46 (2.02) | -43.16 (158.12) / -0.30 (1.77) | -38.64 (213.42) / -0.42 (2.02) | -108.26 (223.30) / -0.97 (1.89) |
| **ΔThal – Mindglide R1+FLAIR Ensemble** | -77.10 (227.31) / -0.70 (2.03) | 5.91 (207.26) / 0.16 (1.71) | -69.32 (273.77) / -0.68 (2.16) | -77.87 (224.01) / -0.72 (2.02) | -103.31 (187.76) / -0.88 (1.86) | -72.20 (257.68) / -0.72 (2.39) | -123.29 (233.97) / -1.05 (1.94) |
| **ΔThal – Mindglide T1+R1+FLAIR Ensemble** | -41.32 (202.53) / -0.41 (1.91) | -18.85 (210.15) / -0.08 (1.80) | -36.15 (257.26) / -0.41 (2.08) | -38.60 (198.33) / -0.39 (1.89) | -21.58 (156.24) / -0.10 (1.75) | -55.37 (237.87) / -0.57 (2.29) | -91.26 (187.42) / -0.84 (1.66) |
| **ΔEDSS** | -0.04 (0.73) / -2.08 (43.93) | - | -0.05 (0.60) / 0.00 (47.14) | -0.09 (0.78) / -4.99 (45.16) | 0.07 (0.43) / 1.20 (11.74) | 0.19 (0.59) / 10.66 (23.67) | -0.08 (0.90) / 0.60 (75.32) |
| **ΔT25FW (s)** | 0.05 (0.84) / 2.50 (13.82) | 0.01 (0.35) / 0.83 (9.64) | 0.19 (0.44) / 5.00 (11.13) | 0.03 (0.76) / 2.04 (13.79) | 0.65 (0.66) / 10.19 (8.48) | 0.08 (1.23) / 2.85 (18.02) | -0.31 (1.44) / -0.66 (16.14) |
| **Δ9HPT (s)** | -0.02 (2.53) / 0.52 (12.08) | -0.20 (2.07) / -1.09 (11.85) | 0.11 (1.65) / 0.77 (8.95) | -0.06 (2.12) / 0.19 (11.33) | -0.16 (2.81) / 0.92 (8.65) | 0.19 (3.71) / 0.24 (12.94) | 0.34 (5.22) / 5.01 (23.11) |
| **ΔSDMT** | 0.49 (7.60) / 2.18 (14.58) | -0.14 (5.01) / -0.61 (8.55) | 2.86 (7.64) / 8.34 (18.86) | 0.96 (7.52) / 2.71 (12.82) | -1.00 (3.92) / -2.74 (8.28) | -4.47 (10.34) / -6.24 (20.45) | 0.83 (6.75) / 7.33 (23.22) |

**Note:** Absolute mean values for delta thalamus volume were scaled to mm^3^ for clarity.

**Table S9. Longitudinal Volume Loss Pairwise Comparisons (n=234)**

| **Method 1** | **Method 2** | **t** | **p** | **p_corr** |
| --- | --- | --- | --- | --- |
| FIRST | DBSegment | -4.60837 | 0.00001 | 0.00005 |
| FIRST | MindGlide T1 | -3.95913 | 0.00010 | 0.00045 |
| FIRST | MindGlide T1-FLAIR-R1 Ensemble | -3.53005 | 0.00050 | 0.00164 |
| FIRST | MindGlide T1-R1 Ensemble | -3.22249 | 0.00145 | 0.00373 |
| FIRST | MindGlide FLAIR | -3.19284 | 0.00160 | 0.00385 |
| FIRST | MindGlide T1-FLAIR Ensemble | -2.87421 | 0.00442 | 0.00885 |
| FIRST | MindGlide R1 | -1.33411 | 0.18347 | 0.23589 |
| FIRST | MindGlide R1-FLAIR Ensemble | -0.98015 | 0.32803 | 0.38094 |
| DBSegment | MindGlide R1-FLAIR Ensemble | 5.24310 | 0.00000 | 0.00001 |
| DBSegment | MindGlide R1 | 4.11797 | 0.00005 | 0.00032 |
| DBSegment | MindGlide T1-FLAIR Ensemble | 3.64085 | 0.00033 | 0.00134 |
| DBSegment | MindGlide T1-R1 Ensemble | 3.23383 | 0.00140 | 0.00373 |
| DBSegment | MindGlide T1-FLAIR-R1 Ensemble | 3.16651 | 0.00175 | 0.00393 |
| DBSegment | MindGlide FLAIR | 3.03083 | 0.00271 | 0.00575 |
| DBSegment | MindGlide T1 | 2.57154 | 0.01075 | 0.02007 |
| MindGlide T1 | MindGlide R1-FLAIR Ensemble | 4.90648 | 0.00000 | 0.00002 |
| MindGlide T1 | MindGlide T1-FLAIR Ensemble | 3.49320 | 0.00057 | 0.00171 |
| MindGlide T1 | MindGlide R1 | 2.44058 | 0.01541 | 0.02642 |
| MindGlide T1 | MindGlide T1-R1 Ensemble | 1.52721 | 0.12807 | 0.17732 |
| MindGlide T1 | MindGlide T1-FLAIR-R1 Ensemble | 1.39655 | 0.16388 | 0.21850 |
| MindGlide T1 | MindGlide FLAIR | 1.23573 | 0.21780 | 0.27037 |
| MindGlide FLAIR | MindGlide R1-FLAIR Ensemble | 4.00546 | 0.00008 | 0.00043 |
| MindGlide FLAIR | MindGlide R1 | 1.80264 | 0.07274 | 0.10911 |
| MindGlide FLAIR | MindGlide T1-FLAIR Ensemble | 1.54878 | 0.12279 | 0.17682 |
| MindGlide FLAIR | MindGlide T1-FLAIR-R1 Ensemble | -0.37508 | 0.70795 | 0.72817 |
| MindGlide FLAIR | MindGlide T1-R1 Ensemble | 0.21136 | 0.83279 | 0.83279 |
| MindGlide R1 | MindGlide T1-FLAIR-R1 Ensemble | -2.55842 | 0.01115 | 0.02007 |
| MindGlide R1 | MindGlide T1-R1 Ensemble | -2.15154 | 0.03246 | 0.05312 |
| MindGlide R1 | MindGlide T1-FLAIR Ensemble | -1.16624 | 0.24471 | 0.29365 |
| MindGlide R1 | MindGlide R1-FLAIR Ensemble | 0.78802 | 0.43149 | 0.45687 |
| MindGlide T1-FLAIR Ensemble | MindGlide R1-FLAIR Ensemble | 3.56649 | 0.00044 | 0.00158 |
| MindGlide T1-FLAIR Ensemble | MindGlide T1-FLAIR-R1 Ensemble | -2.03808 | 0.04267 | 0.06679 |
| MindGlide T1-FLAIR Ensemble | MindGlide T1-R1 Ensemble | -0.83355 | 0.40539 | 0.44571 |
| MindGlide T1-R1 Ensemble | MindGlide R1-FLAIR Ensemble | 5.32195 | 0.00000 | 0.00001 |
| MindGlide T1-R1 Ensemble | MindGlide T1-FLAIR-R1 Ensemble | -0.82792 | 0.40856 | 0.44571 |
| MindGlide R1-FLAIR Ensemble | MindGlide T1-FLAIR-R1 Ensemble | -5.10963 | 0.00000 | 0.00001 |

**Table S10. Longitudinal Mixed Effects Results**

| **ΔClinical Outcome** | **Predictor (Δ Thalamus Volume)** | **n** | **β** | **SE** | **95%CI** | **p** | **p_corr** | **R2** |
| --- | --- | --- | --- | --- | --- | --- | --- | --- |
| **ΔEDSS** | FIRST | 221 | 0.517 | 0.156 | [0.212, 0.822] | 0.0009 | 0.0315 | 0.067 |
|  | DBSegment | 221 | 0.595 | 0.250 | [0.105, 1.086] | 0.0173 | 0.0690 | 0.041 |
|  | MindGlide T1 | 221 | 0.579 | 0.220 | [0.147, 1.011] | 0.0086 | 0.0442 | 0.055 |
|  | MindGlide FLAIR | 221 | 0.617 | 0.241 | [0.144, 1.089] | 0.0105 | 0.0474 | 0.056 |
|  | MindGlide R1 | 221 | 0.614 | 0.209 | [0.203, 1.024] | 0.0034 | 0.0315 | 0.059 |
|  | MindGlide T1-FLAIR Ensemble | 221 | 0.604 | 0.213 | [0.187, 1.020] | 0.0045 | 0.0315 | 0.060 |
|  | MindGlide T1-R1 Ensemble | 221 | 0.648 | 0.228 | [0.201, 1.096] | 0.0045 | 0.0315 | 0.060 |
|  | MindGlide R1-FLAIR Ensemble | 221 | 0.657 | 0.225 | [0.215, 1.098] | 0.0036 | 0.0315 | 0.064 |
|  | MindGlide T1-FLAIR-R1 Ensemble | 221 | 0.698 | 0.250 | [0.208, 1.188] | 0.0052 | 0.0315 | 0.060 |
| **ΔT25FW (s)** | FIRST | 219 | 0.285 | 0.190 | [-0.089, 0.659] | 0.1340 | 0.4824 | 0.043 |
|  | DBSegment | 219 | 0.458 | 0.393 | [-0.315, 1.232] | 0.2443 | 0.7102 | 0.039 |
|  | MindGlide T1 | 219 | 0.275 | 0.267 | [-0.252, 0.801] | 0.3046 | 0.7102 | 0.038 |
|  | MindGlide FLAIR | 219 | 0.043 | 0.270 | [-0.489, 0.575] | 0.8736 | 0.9250 | 0.033 |
|  | MindGlide R1 | 219 | 0.264 | 0.252 | [-0.232, 0.761] | 0.2950 | 0.7102 | 0.038 |
|  | MindGlide T1-FLAIR Ensemble | 219 | 0.147 | 0.254 | [-0.353, 0.647] | 0.5623 | 0.7735 | 0.034 |
|  | MindGlide T1-R1 Ensemble | 219 | 0.140 | 0.267 | [-0.387, 0.667] | 0.6016 | 0.7735 | 0.034 |
|  | MindGlide R1-FLAIR Ensemble | 219 | 0.095 | 0.256 | [-0.410, 0.599] | 0.7117 | 0.7764 | 0.033 |
|  | MindGlide T1-FLAIR-R1 Ensemble | 219 | 0.216 | 0.289 | [-0.354, 0.786] | 0.4555 | 0.7492 | 0.035 |
| **Δ9HPT (s)** | FIRST | 222 | 0.206 | 0.529 | [-0.832, 1.243] | 0.6976 | 0.7764 | 0.035 |
|  | DBSegment | 222 | -0.357 | 0.875 | [-2.072, 1.357] | 0.6829 | 0.7764 | 0.035 |
|  | MindGlide T1 | 222 | -0.536 | 0.707 | [-1.921, 0.850] | 0.4485 | 0.7492 | 0.037 |
|  | MindGlide FLAIR | 222 | -0.768 | 0.631 | [-2.004, 0.468] | 0.2235 | 0.7102 | 0.039 |
|  | MindGlide R1 | 222 | -0.536 | 0.666 | [-1.841, 0.769] | 0.4207 | 0.7492 | 0.037 |
|  | MindGlide T1-FLAIR Ensemble | 222 | -0.657 | 0.624 | [-1.881, 0.566] | 0.2924 | 0.7102 | 0.038 |
|  | MindGlide T1-R1 Ensemble | 222 | -0.412 | 0.693 | [-1.770, 0.947] | 0.5527 | 0.7735 | 0.036 |
|  | MindGlide R1-FLAIR Ensemble | 222 | -0.443 | 0.574 | [-1.569, 0.683] | 0.4404 | 0.7492 | 0.036 |
|  | MindGlide T1-FLAIR-R1 Ensemble | 222 | -0.764 | 0.767 | [-2.267, 0.739] | 0.3190 | 0.7102 | 0.038 |
| **ΔSDMT** | FIRST | 220 | -0.120 | 1.705 | [-3.482, 3.242] | 0.9440 | 0.9708 | 0.056 |
|  | DBSegment | 220 | -1.921 | 3.561 | [-8.940, 5.099] | 0.5902 | 0.7735 | 0.057 |
|  | MindGlide T1 | 220 | 0.087 | 2.382 | [-4.609, 4.784] | 0.9708 | 0.9708 | 0.056 |
|  | MindGlide FLAIR | 220 | -2.322 | 2.405 | [-7.063, 2.418] | 0.3353 | 0.7102 | 0.060 |
|  | MindGlide R1 | 220 | -1.600 | 2.254 | [-6.042, 2.843] | 0.4786 | 0.7492 | 0.058 |
|  | MindGlide T1-FLAIR Ensemble | 220 | -0.979 | 2.262 | [-5.438, 3.480] | 0.6656 | 0.7764 | 0.057 |
|  | MindGlide T1-R1 Ensemble | 220 | -1.356 | 2.386 | [-6.060, 3.347] | 0.5703 | 0.7735 | 0.057 |
|  | MindGlide R1-FLAIR Ensemble | 220 | -1.688 | 2.293 | [-6.208, 2.831] | 0.4623 | 0.7492 | 0.058 |
|  | MindGlide T1-FLAIR-R1 Ensemble | 220 | -1.199 | 2.577 | [-6.279, 3.880] | 0.6421 | 0.7764 | 0.057 |

1. **Supplementary Sensitivity Analyses: Clinical Associations for RRMS Cohort**

**Table S11. Mass Univariate Analysis Results – Cross-sectional Outcome Analysis, RRMS Cohort**

| **Clinical Outcome** | **Predictor (thalamus volume in mL)** | **n** | **β** | **SE** | **t** | **p** | **p_corr** | **R2** |
| --- | --- | --- | --- | --- | --- | --- | --- | --- |
| **EDSS** | FIRST | 201 | -0,190 | 0,055 | -3,455 | 0,0006 | 0,0010 | 0,226 |
|  | DBSegment | 201 | -0,291 | 0,090 | -3,226 | 0,0013 | 0,0020 | 0,209 |
|  | MindGlide T1 | 201 | -0,230 | 0,064 | -3,598 | 0,0003 | 0,0007 | 0,220 |
|  | MindGlide FLAIR | 201 | -0,250 | 0,068 | -3,681 | 0,0002 | 0,0005 | 0,227 |
|  | MindGlide R1 | 201 | -0,225 | 0,064 | -3,539 | 0,0004 | 0,0008 | 0,218 |
|  | MindGlide T1-FLAIR Ensemble | 201 | -0,233 | 0,063 | -3,690 | 0,0002 | 0,0005 | 0,224 |
|  | MindGlide T1-R1 Ensemble | 201 | -0,221 | 0,062 | -3,583 | 0,0003 | 0,0007 | 0,219 |
|  | MindGlide R1-FLAIR Ensemble | 201 | -0,230 | 0,061 | -3,736 | 0,0002 | 0,0005 | 0,224 |
|  | MindGlide T1-FLAIR-R1 Ensemble | 201 | -0,238 | 0,065 | -3,660 | 0,0003 | 0,0006 | 0,222 |
| **T25FW log(s)** | FIRST | 203 | -0,027 | 0,009 | -3,110 | 0,0021 | 0,0028 | 0,121 |
|  | DBSegment | 203 | -0,033 | 0,016 | -2,046 | 0,0421 | 0,0421 | 0,098 |
|  | MindGlide T1 | 203 | -0,031 | 0,011 | -2,760 | 0,0063 | 0,0071 | 0,113 |
|  | MindGlide FLAIR | 203 | -0,033 | 0,012 | -2,864 | 0,0046 | 0,0055 | 0,115 |
|  | MindGlide R1 | 203 | -0,029 | 0,011 | -2,603 | 0,0099 | 0,0102 | 0,109 |
|  | MindGlide T1-FLAIR Ensemble | 203 | -0,030 | 0,011 | -2,741 | 0,0067 | 0,0073 | 0,112 |
|  | MindGlide T1-R1 Ensemble | 203 | -0,029 | 0,011 | -2,659 | 0,0085 | 0,0091 | 0,110 |
|  | MindGlide R1-FLAIR Ensemble | 203 | -0,029 | 0,011 | -2,650 | 0,0087 | 0,0091 | 0,110 |
|  | MindGlide T1-FLAIR-R1 Ensemble | 203 | -0,031 | 0,011 | -2,760 | 0,0063 | 0,0071 | 0,113 |
| **9HPT log(s)** | FIRST | 203 | -0,039 | 0,008 | -5,089 | 0,0000 | 0,0000 | 0,249 |
|  | DBSegment | 203 | -0,048 | 0,015 | -3,299 | 0,0011 | 0,0020 | 0,195 |
|  | MindGlide T1 | 203 | -0,042 | 0,010 | -4,274 | 0,0000 | 0,0001 | 0,222 |
|  | MindGlide FLAIR | 203 | -0,045 | 0,010 | -4,446 | 0,0000 | 0,0001 | 0,227 |
|  | MindGlide R1 | 203 | -0,045 | 0,010 | -4,538 | 0,0000 | 0,0001 | 0,230 |
|  | MindGlide T1-FLAIR Ensemble | 203 | -0,042 | 0,010 | -4,358 | 0,0000 | 0,0001 | 0,225 |
|  | MindGlide T1-R1 Ensemble | 203 | -0,042 | 0,010 | -4,410 | 0,0000 | 0,0001 | 0,226 |
|  | MindGlide R1-FLAIR Ensemble | 203 | -0,042 | 0,010 | -4,446 | 0,0000 | 0,0001 | 0,227 |
|  | MindGlide T1-FLAIR-R1 Ensemble | 203 | -0,044 | 0,010 | -4,372 | 0,0000 | 0,0001 | 0,225 |
| **SDMT** | FIRST | 201 | 2,028 | 0,580 | 3,495 | 0,0006 | 0,0011 | 0,168 |
|  | DBSegment | 201 | 3,222 | 1,074 | 3,001 | 0,0030 | 0,0038 | 0,155 |
|  | MindGlide T1 | 201 | 2,349 | 0,751 | 3,127 | 0,0020 | 0,0027 | 0,158 |
|  | MindGlide FLAIR | 201 | 2,493 | 0,766 | 3,253 | 0,0013 | 0,0020 | 0,161 |
|  | MindGlide R1 | 201 | 2,451 | 0,752 | 3,259 | 0,0013 | 0,0020 | 0,161 |
|  | MindGlide T1-FLAIR Ensemble | 201 | 2,386 | 0,732 | 3,261 | 0,0013 | 0,0020 | 0,162 |
|  | MindGlide T1-R1 Ensemble | 201 | 2,289 | 0,726 | 3,154 | 0,0019 | 0,0026 | 0,159 |
|  | MindGlide R1-FLAIR Ensemble | 201 | 2,260 | 0,721 | 3,134 | 0,0020 | 0,0027 | 0,158 |
|  | MindGlide T1-FLAIR-R1 Ensemble | 201 | 2,424 | 0,758 | 3,200 | 0,0016 | 0,0023 | 0,160 |
| **Disease duration (years)** | FIRST | 202 | -1,474 | 0,365 | -4,040 | 0,0001 | 0,0002 | 0,346 |
|  | DBSegment | 202 | -2,057 | 0,708 | -2,905 | 0,0037 | 0,0045 | 0,320 |
|  | MindGlide T1 | 202 | -2,071 | 0,483 | -4,288 | 0,0000 | 0,0001 | 0,358 |
|  | MindGlide FLAIR | 202 | -2,101 | 0,490 | -4,289 | 0,0000 | 0,0001 | 0,357 |
|  | MindGlide R1 | 202 | -2,091 | 0,482 | -4,338 | 0,0000 | 0,0001 | 0,359 |
|  | MindGlide T1-FLAIR Ensemble | 202 | -2,019 | 0,472 | -4,274 | 0,0000 | 0,0001 | 0,358 |
|  | MindGlide T1-R1 Ensemble | 202 | -2,034 | 0,463 | -4,390 | 0,0000 | 0,0001 | 0,360 |
|  | MindGlide R1-FLAIR Ensemble | 202 | -2,043 | 0,461 | -4,435 | 0,0000 | 0,0001 | 0,362 |
|  | MindGlide T1-FLAIR-R1 Ensemble | 202 | -2,095 | 0,487 | -4,297 | 0,0000 | 0,0001 | 0,358 |

***Note:*** EDSS and disease duration OLS models were fitted with robust standard errors (HC3) due to heteroscedasticity. All remaining outcomes were fitted with standard OLS.


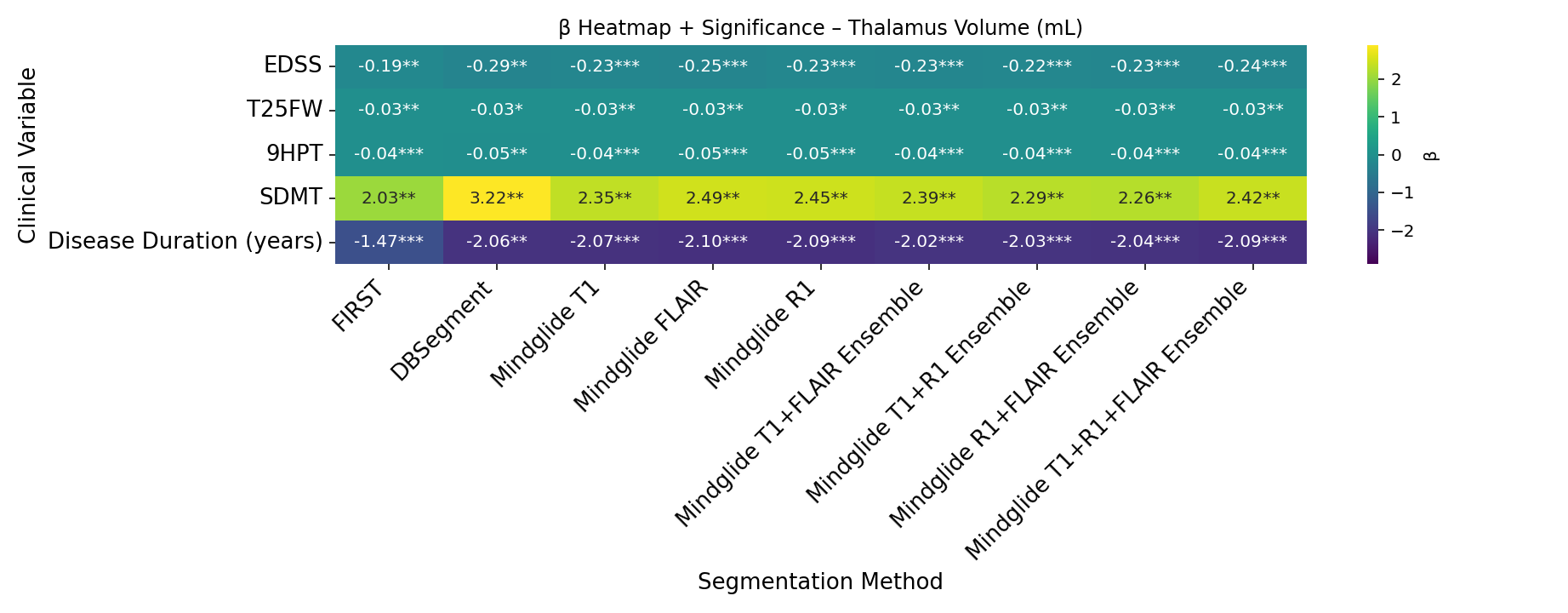
**Figure S3.** Summary of cross-sectional analysis OLS results with the RRMS-only cohort (n=201-203)

**Table S12. Longitudinal Mixed Effects Results, RRMS Cohort**

| **ΔClinical Outcome** | **Predictor (Δ Thalamus Volume)** | **n** | **β** | **se** | **CI95%** | **p** | **p_corr** | **R^2^** |
| --- | --- | --- | --- | --- | --- | --- | --- | --- |
| **ΔEDSS** | FIRST | 155 | 0,411 | 0,215 | [-0.010, 0.833] | 0,0558 | 0,4678 | 0,061 |
|  | DBSegment | 155 | 0,400 | 0,269 | [-0.128, 0.928] | 0,1376 | 0,4952 | 0,046 |
|  | MindGlide T1 | 155 | 0,450 | 0,283 | [-0.105, 1.006] | 0,1123 | 0,4952 | 0,055 |
|  | MindGlide FLAIR | 155 | 0,492 | 0,322 | [-0.139, 1.122] | 0,1266 | 0,4952 | 0,057 |
|  | MindGlide R1 | 155 | 0,619 | 0,265 | [0.099, 1.139] | 0,0196 | 0,4678 | 0,071 |
|  | MindGlide T1-FLAIR Ensemble | 155 | 0,466 | 0,276 | [-0.074, 1.007] | 0,0910 | 0,4678 | 0,058 |
|  | MindGlide T1-R1 Ensemble | 155 | 0,540 | 0,282 | [-0.013, 1.093] | 0,0556 | 0,4678 | 0,063 |
|  | MindGlide R1-FLAIR Ensemble | 155 | 0,540 | 0,293 | [-0.034, 1.114] | 0,0654 | 0,4678 | 0,063 |
|  | MindGlide T1-FLAIR-R1 Ensemble | 155 | 0,555 | 0,322 | [-0.076, 1.186] | 0,0846 | 0,4678 | 0,060 |
| **ΔT25FW (s)** | FIRST | 158 | 0,012 | 0,219 | [-0.420, 0.444] | 0,9568 | 0,9841 | 0,042 |
|  | DBSegment | 158 | 0,078 | 0,432 | [-0.776, 0.931] | 0,8577 | 0,9841 | 0,043 |
|  | MindGlide T1 | 158 | -0,037 | 0,287 | [-0.603, 0.530] | 0,8978 | 0,9841 | 0,042 |
|  | MindGlide FLAIR | 158 | 0,031 | 0,295 | [-0.551, 0.613] | 0,9159 | 0,9841 | 0,042 |
|  | MindGlide R1 | 158 | 0,180 | 0,269 | [-0.352, 0.712] | 0,5045 | 0,9841 | 0,045 |
|  | MindGlide T1-FLAIR Ensemble | 158 | -0,028 | 0,275 | [-0.572, 0.516] | 0,9191 | 0,9841 | 0,042 |
|  | MindGlide T1-R1 Ensemble | 158 | -0,142 | 0,279 | [-0.693, 0.410] | 0,6129 | 0,9841 | 0,044 |
|  | MindGlide R1-FLAIR Ensemble | 158 | -0,003 | 0,272 | [-0.541, 0.534] | 0,9900 | 0,9900 | 0,042 |
|  | MindGlide T1-FLAIR-R1 Ensemble | 158 | -0,043 | 0,309 | [-0.654, 0.567] | 0,8891 | 0,9841 | 0,042 |
| **Δ9HPT (s)** | FIRST | 157 | 0,831 | 0,480 | [-0.110, 1.773] | 0,0836 | 0,4678 | 0,104 |
|  | DBSegment | 157 | 0,061 | 1,047 | [-1.991, 2.113] | 0,9535 | 0,9841 | 0,093 |
|  | MindGlide T1 | 157 | 0,348 | 0,735 | [-1.093, 1.789] | 0,6360 | 0,9841 | 0,094 |
|  | MindGlide FLAIR | 157 | -0,077 | 0,618 | [-1.289, 1.135] | 0,9008 | 0,9841 | 0,093 |
|  | MindGlide R1 | 157 | -0,054 | 0,617 | [-1.264, 1.156] | 0,9303 | 0,9841 | 0,093 |
|  | MindGlide T1-FLAIR Ensemble | 157 | 0,190 | 0,607 | [-0.999, 1.378] | 0,7545 | 0,9841 | 0,093 |
|  | MindGlide T1-R1 Ensemble | 157 | 0,284 | 0,622 | [-0.934, 1.502] | 0,6476 | 0,9841 | 0,093 |
|  | MindGlide R1-FLAIR Ensemble | 157 | 0,244 | 0,516 | [-0.767, 1.256] | 0,6360 | 0,9841 | 0,093 |
|  | MindGlide T1-FLAIR-R1 Ensemble | 157 | 0,277 | 0,701 | [-1.098, 1.652] | 0,6927 | 0,9841 | 0,093 |
| **ΔSDMT** | FIRST | 156 | 0,192 | 2,189 | [-4.134, 4.518] | 0,9303 | 0,9841 | 0,022 |
|  | DBSegment | 156 | -3,711 | 4,361 | [-12.328, 4.905] | 0,3961 | 0,9841 | 0,026 |
|  | MindGlide T1 | 156 | -0,176 | 2,877 | [-5.861, 5.508] | 0,9513 | 0,9841 | 0,022 |
|  | MindGlide FLAIR | 156 | -1,965 | 2,950 | [-7.794, 3.865] | 0,5065 | 0,9841 | 0,025 |
|  | MindGlide R1 | 156 | -1,609 | 2,708 | [-6.960, 3.742] | 0,5533 | 0,9841 | 0,024 |
|  | MindGlide T1-FLAIR Ensemble | 156 | -1,715 | 2,757 | [-7.162, 3.733] | 0,5349 | 0,9841 | 0,024 |
|  | MindGlide T1-R1 Ensemble | 156 | -1,389 | 2,802 | [-6.925, 4.148] | 0,6209 | 0,9841 | 0,023 |
|  | MindGlide R1-FLAIR Ensemble | 156 | -2,539 | 2,721 | [-7.916, 2.837] | 0,3522 | 0,9841 | 0,027 |
|  | MindGlide T1-FLAIR-R1 Ensemble | 156 | -1,496 | 3,097 | [-7.615, 4.624] | 0,6298 | 0,9841 | 0,023 |


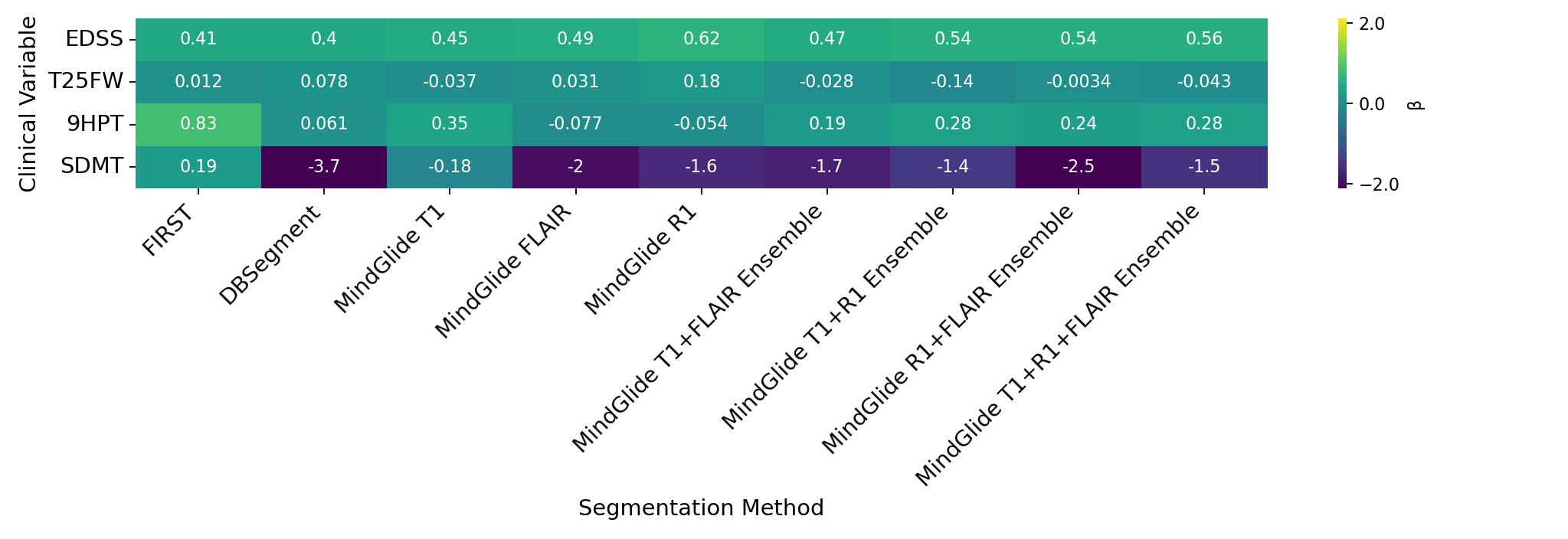
**Figure S4.** Summary of longitudinal analysis OLS results between thalamus volume change and clinical outcome change with the RRMS-only cohort (n=155-157)

1. **Supplementary Sensitivity Analyses: ΔEDSS**

**Table S13. Longitudinal Mixed Effects Results, ΔThalamus Volume 1-99% Winsorization**

| ΔClinical Outcome | Predictor (Δ Thalamus Volume) | n | β | se | CI95% | p | p_corr | R^2^ |
| --- | --- | --- | --- | --- | --- | --- | --- | --- |
| ΔEDSS | FIRST | 221 | 0,533 | 0,178 | [0.185, 0.881] | 0,0027 | 0,0441 | 0,064 |
|  | DBSegment | 221 | 0,655 | 0,273 | [0.120, 1.189] | 0,0164 | 0,0655 | 0,041 |
|  | MindGlide T1 | 221 | 0,598 | 0,246 | [0.116, 1.079] | 0,0151 | 0,0655 | 0,052 |
|  | MindGlide FLAIR | 221 | 0,618 | 0,251 | [0.126, 1.110] | 0,0138 | 0,0655 | 0,055 |
|  | MindGlide R1 | 221 | 0,661 | 0,231 | [0.208, 1.113] | 0,0042 | 0,0441 | 0,059 |
|  | MindGlide T1-FLAIR Ensemble | 221 | 0,621 | 0,239 | [0.153, 1.090] | 0,0093 | 0,0560 | 0,056 |
|  | MindGlide T1-R1 Ensemble | 221 | 0,727 | 0,258 | [0.220, 1.233] | 0,0049 | 0,0441 | 0,061 |
|  | MindGlide R1-FLAIR Ensemble | 221 | 0,696 | 0,242 | [0.221, 1.171] | 0,0041 | 0,0441 | 0,064 |
|  | MindGlide T1-FLAIR-R1 Ensemble | 221 | 0,725 | 0,273 | [0.189, 1.261] | 0,0080 | 0,0560 | 0,059 |

**Table S14. Longitudinal Mixed Effects Results, ΔThalamus Volume 5-95% Winsorization**

| ΔClinical Outcome | Predictor (Δ Thalamus Volume) | n | β | se | CI95% | p | q | R^2^ |
| --- | --- | --- | --- | --- | --- | --- | --- | --- |
| ΔEDSS | FIRST | 221 | 0,596 | 0,203 | [0.199, 0.994] | 0,0033 | 0,0785 | 0,065 |
|  | DBSegment | 221 | 0,869 | 0,466 | [-0.045, 1.783] | 0,0625 | 0,2501 | 0,039 |
|  | MindGlide T1 | 221 | 0,591 | 0,306 | [-0.009, 1.190] | 0,0534 | 0,2402 | 0,045 |
|  | MindGlide FLAIR | 221 | 0,638 | 0,302 | [0.045, 1.230] | 0,0350 | 0,1798 | 0,049 |
|  | MindGlide R1 | 221 | 0,778 | 0,273 | [0.243, 1.312] | 0,0044 | 0,0785 | 0,063 |
|  | MindGlide T1-FLAIR Ensemble | 221 | 0,665 | 0,288 | [0.100, 1.230] | 0,0210 | 0,1261 | 0,052 |
|  | MindGlide T1-R1 Ensemble | 221 | 0,764 | 0,299 | [0.179, 1.349] | 0,0105 | 0,0946 | 0,057 |
|  | MindGlide R1-FLAIR Ensemble | 221 | 0,767 | 0,284 | [0.210, 1.324] | 0,0070 | 0,0837 | 0,063 |
|  | MindGlide T1-FLAIR-R1 Ensemble | 221 | 0,759 | 0,315 | [0.141, 1.376] | 0,0160 | 0,1153 | 0,055 |

**Table S15. Longitudinal Ordinal Logistic Regression, ΔEDSS**

| ΔClinical Outcome | Predictor (Δ Thalamus Volume) | β | SE | OR per unit | z | p | p_corr |
| --- | --- | --- | --- | --- | --- | --- | --- |
| **ΔEDSS** | FIRST | 1,019 | 0,411 | 2,769 | 2,478 | 0,0132 | 0,0882 |
|  | DBSegment | 1,589 | 0,781 | 4,898 | 2,034 | 0,0419 | 0,0882 |
|  | MindGlide T1 | 0,954 | 0,544 | 2,597 | 1,754 | 0,0795 | 0,0894 |
|  | MindGlide FLAIR | 0,874 | 0,572 | 2,397 | 1,529 | 0,1262 | 0,1262 |
|  | MindGlide R1 | 1,167 | 0,547 | 3,213 | 2,134 | 0,0328 | 0,0882 |
|  | MindGlide T1-FLAIR Ensemble | 0,970 | 0,527 | 2,637 | 1,839 | 0,0659 | 0,0882 |
|  | MindGlide T1-R1 Ensemble | 1,022 | 0,561 | 2,778 | 1,821 | 0,0686 | 0,0882 |
|  | MindGlide R1-FLAIR Ensemble | 1,017 | 0,544 | 2,765 | 1,870 | 0,0614 | 0,0882 |
|  | MindGlide T1-FLAIR-R1 Ensemble | 1,138 | 0,603 | 3,122 | 1,888 | 0,0591 | 0,0882 |

***Note:*** ΔEDSS was divided into 5 categories: strong improvement (ΔEDSS ≤ -1.5, n=11), slight improvement (-1.5 < ΔEDSS < 0, n=54), stable (ΔEDSS = 0, n=97), slight worsening (0 < ΔEDSS < 1.5, n=44), strong worsening (ΔEDSS ≥ 1.5, n=15).


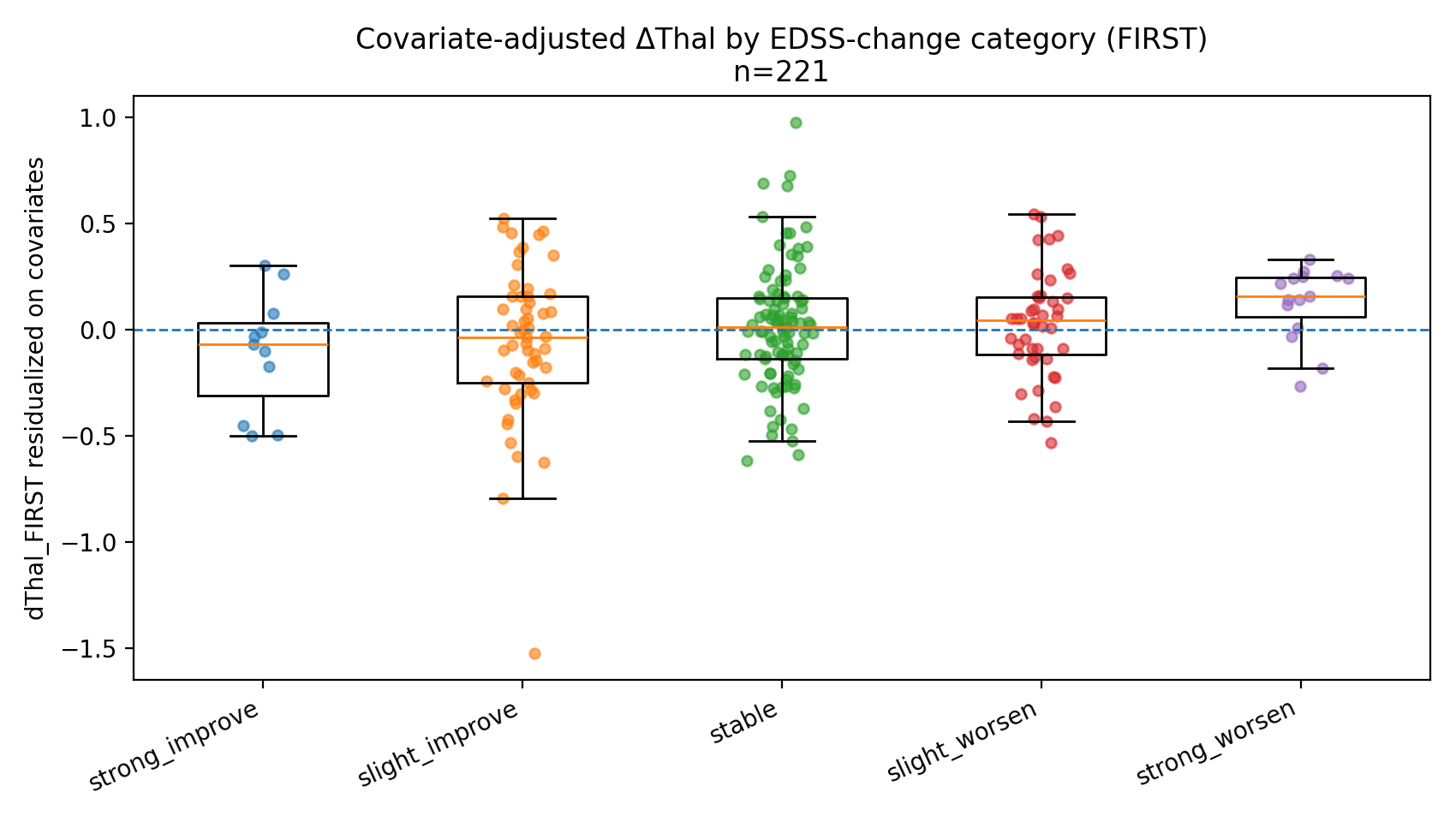

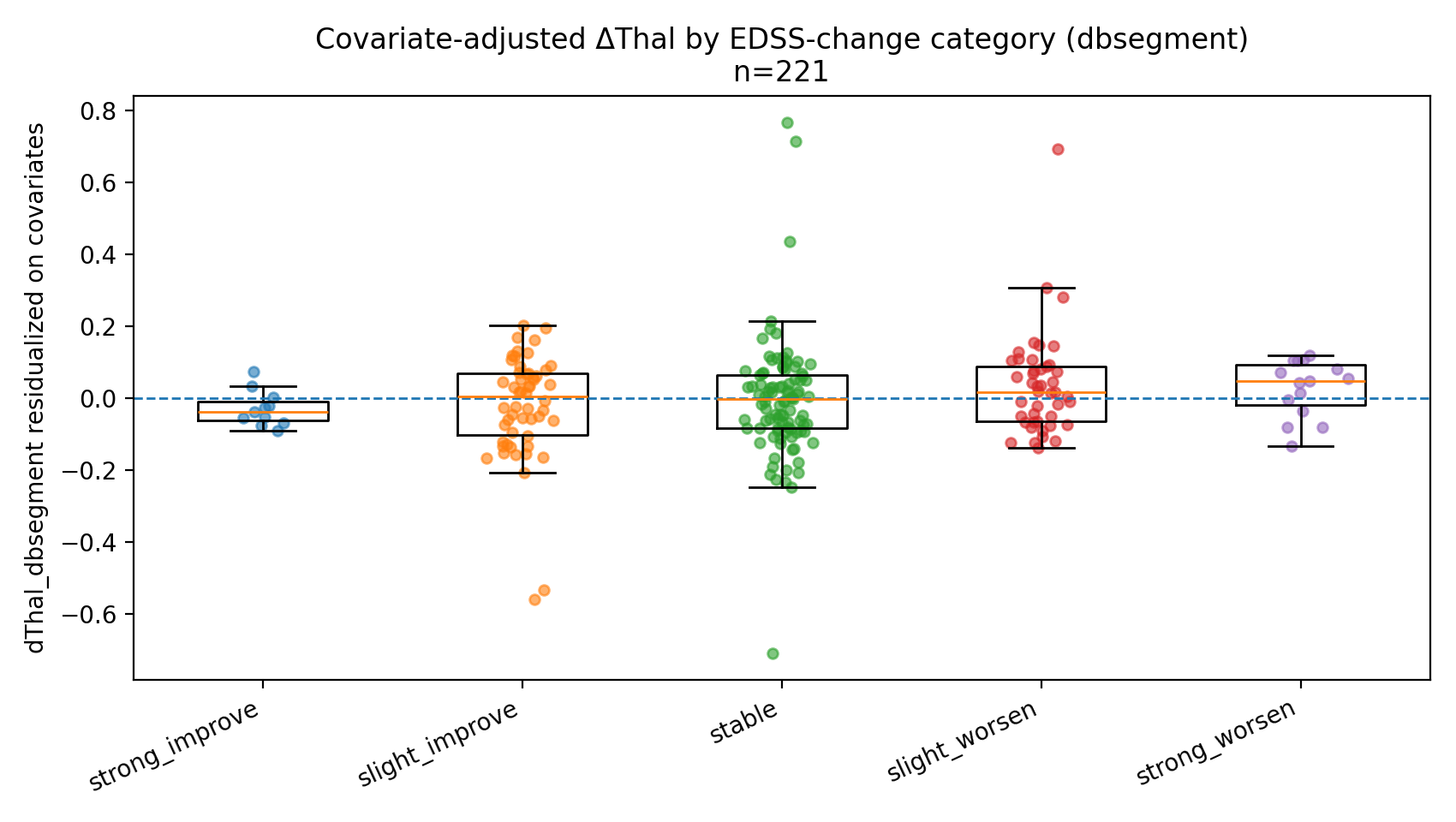

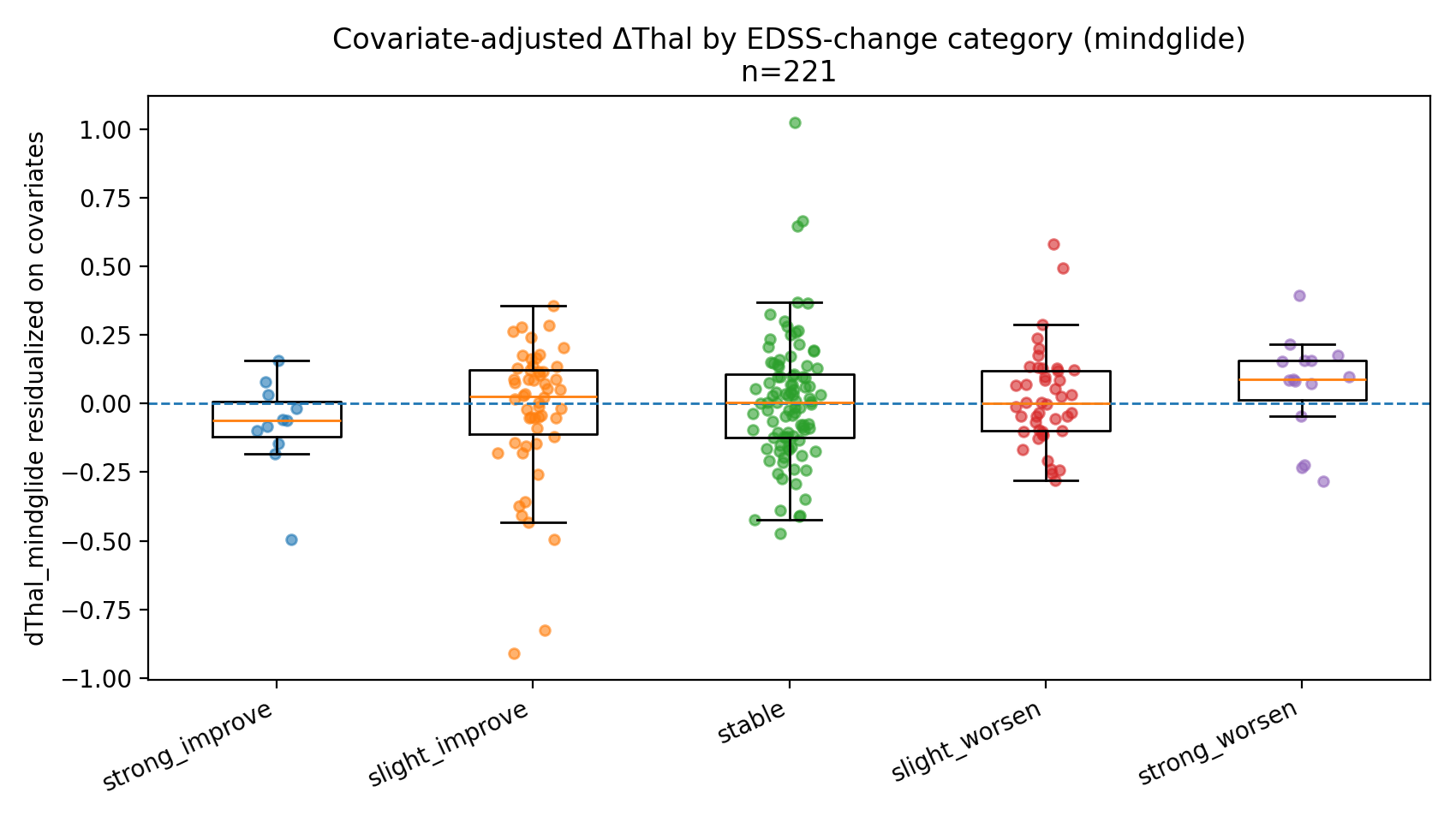


**Figure S5.** Boxplots illustrating the association between Δ Thalamus Volume (residualized on covariates) and categorical ΔEDSS for FIRST, DBSegment and MindGlide T1 respectively. Remaining MindGlide ensemble labels follow the same pattern as MindGlide T1, and were omitted for brevity.

**SM.E – MRI-derived PBVC Associations**


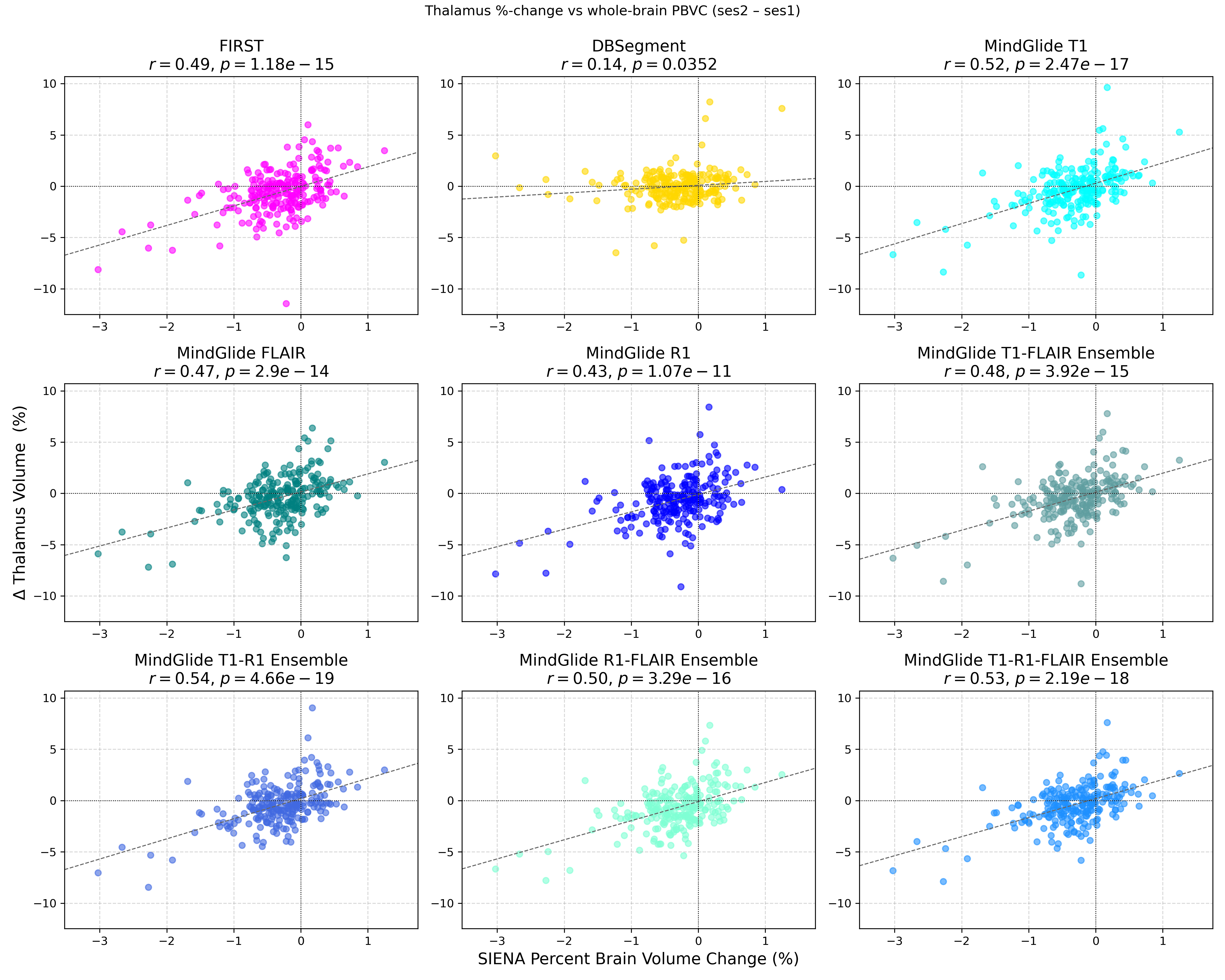


**Figure S6.** Association of algorithm-derived thalamus volume loss (in %) with SIENA-derived PBVC (in %) computed between 1-year follow-up and baseline. A total of n = 234 participants were included; no stratification for disease diagnosis is shown.

**Table S16. SIENA PBVC vs Thalamus Volume Change Pairwise Comparisons (n=234)**

| **Method 1** | **Method 2** | **z** | **p** | **p_corr** |
| --- | --- | --- | --- | --- |
| FIRST | DBSegment | 4.18161 | 0.00004 | 0.00021 |
| FIRST | MindGlide T1 | -0.43362 | 0.66497 | 0.77152 |
| FIRST | MindGlide FLAIR | 0.36837 | 0.71293 | 0.77152 |
| FIRST | MindGlide R1 | 0.93059 | 0.35304 | 0.60958 |
| FIRST | MindGlide T1-FLAIR Ensemble | 0.15271 | 0.87876 | 0.87984 |
| FIRST | MindGlide T1-R1 Ensemble | -0.86224 | 0.38945 | 0.60958 |
| FIRST | MindGlide R1-FLAIR Ensemble | -0.15134 | 0.87984 | 0.87984 |
| FIRST | MindGlide T1-R1-FLAIR Ensemble | -0.70914 | 0.47895 | 0.68969 |
| DBSegment | MindGlide T1 | -5.62391 | 0.00000 | 0.00000 |
| DBSegment | MindGlide FLAIR | -4.30148 | 0.00003 | 0.00015 |
| DBSegment | MindGlide R1 | -3.05097 | 0.00255 | 0.01146 |
| DBSegment | MindGlide T1-FLAIR Ensemble | -4.72502 | 0.00000 | 0.00004 |
| DBSegment | MindGlide T1-R1 Ensemble | -5.10641 | 0.00000 | 0.00001 |
| DBSegment | MindGlide R1-FLAIR Ensemble | -4.47834 | 0.00001 | 0.00009 |
| DBSegment | MindGlide T1-R1-FLAIR Ensemble | -5.23420 | 0.00000 | 0.00001 |
| MindGlide T1 | MindGlide FLAIR | 1.25959 | 0.20909 | 0.39616 |
| MindGlide T1 | MindGlide R1 | 1.28480 | 0.20015 | 0.39616 |
| MindGlide T1 | MindGlide T1-FLAIR Ensemble | 1.36987 | 0.17206 | 0.39616 |
| MindGlide T1 | MindGlide T1-R1 Ensemble | -0.62257 | 0.53418 | 0.71224 |
| MindGlide T1 | MindGlide R1-FLAIR Ensemble | 0.38417 | 0.70120 | 0.77152 |
| MindGlide T1 | MindGlide T1-R1-FLAIR Ensemble | -0.56496 | 0.57265 | 0.71406 |
| MindGlide FLAIR | MindGlide R1 | 0.65413 | 0.51368 | 0.71125 |
| MindGlide FLAIR | MindGlide T1-FLAIR Ensemble | -0.56119 | 0.57521 | 0.71406 |
| MindGlide FLAIR | MindGlide T1-R1 Ensemble | -1.48215 | 0.13966 | 0.37400 |
| MindGlide FLAIR | MindGlide R1-FLAIR Ensemble | -0.75740 | 0.44958 | 0.67437 |
| MindGlide FLAIR | MindGlide T1-R1-FLAIR Ensemble | -2.00758 | 0.04585 | 0.15006 |
| MindGlide R1 | MindGlide T1-FLAIR Ensemble | -0.87349 | 0.38330 | 0.60958 |
| MindGlide R1 | MindGlide T1-R1 Ensemble | -2.25339 | 0.02517 | 0.10069 |
| MindGlide R1 | MindGlide R1-FLAIR Ensemble | -1.26894 | 0.20574 | 0.39616 |
| MindGlide R1 | MindGlide T1-R1-FLAIR Ensemble | -2.01867 | 0.04468 | 0.15006 |
| MindGlide T1-FLAIR Ensemble | MindGlide T1-R1 Ensemble | -1.46074 | 0.14545 | 0.37400 |
| MindGlide T1-FLAIR Ensemble | MindGlide R1-FLAIR Ensemble | -0.50744 | 0.61233 | 0.73480 |
| MindGlide T1-FLAIR Ensemble | MindGlide T1-R1-FLAIR Ensemble | -1.88684 | 0.06044 | 0.18131 |
| MindGlide T1-R1 Ensemble | MindGlide R1-FLAIR Ensemble | 1.32494 | 0.18650 | 0.39616 |
| MindGlide T1-R1 Ensemble | MindGlide T1-R1-FLAIR Ensemble | 0.34733 | 0.72866 | 0.77152 |
| MindGlide R1-FLAIR Ensemble | MindGlide T1-R1-FLAIR Ensemble | -0.88961 | 0.37460 | 0.60958 |
